# Spray‐Assisted Fabrication of Cellulose Photonic Pigments on Superhydrophobic Surfaces

**DOI:** 10.1002/adma.202416607

**Published:** 2025-01-29

**Authors:** Jianing Song, Richard M. Parker, Bruno Frka‐Petesic, Tao Deng, Luqing Xu, Xu Deng, Silvia Vignolini, Qingchen Shen

**Affiliations:** ^1^ Yusuf Hamied Department of Chemistry University of Cambridge Lensfield Road Cambridge CB2 1EW UK; ^2^ Shenzhen Institute for Advanced Study University of Electronic Science and Technology of China Shenzhen 518110 P. R. China; ^3^ Institute of Fundamental and Frontier Sciences University of Electronic Science and Technology of China Chengdu 611731 P. R. China; ^4^ International Institute for Sustainability with Knotted Chiral Meta Matter (WPI‐SKCM^2^) Hiroshima University 1‐3‐1 Kagamiyama Higashi‐Hiroshima Hiroshima 739–8526 Japan; ^5^ State Key Laboratory of Metal Matrix Composites School of Materials Science and Engineering Shanghai Jiao Tong University Shanghai 200240 P. R. China; ^6^ Max Planck Institute of Colloids and Interfaces Science Park Golm 14476 Potsdam Germany

**Keywords:** cellulose nanocrystals, cholesteric self‐assembly, photonic pigments, superhydrophobic surfaces, sustainable materials

## Abstract

Photonic pigments, especially those based on naturally‐derived building blocks like cellulose nanocrystals (CNCs), are emerging as a promising sustainable alternative to absorption‐based colorants. However, the proposed manufacturing methods for CNC pigments, via either grinding films or emulsion‐based production, usually require several processing steps. This limits their commercialization by increasing the costs, timescales, and environmental impacts of production. Toward addressing these challenges, it is reported that photonic pigments can be produced in a single process by drying microdroplets of aqueous CNC suspension on a superhydrophobic surface. Such liquid‐repellent substrate ensures the microdroplets maintain a near‐spherical shape, enabling the radial self‐organization of the cholesteric phase. Upon drying under ambient conditions, the CNC mesophase becomes kinetically arrested, after which the strong capillary forces induced by water evaporation result in extensive buckling of the microparticle. This buckling, coupled with prior tuning of the CNC formulation, enables photonic pigments with adjustable color across the visible spectrum. Importantly, the elimination of an emulsifying oil phase to create microdroplets enables a much faster drying time (≈40 min) and improved color stability (e.g., polar solvents, elevated temperatures), while the reduction in reagents (e.g., oils, surfactants) and post‐processing steps (e.g., solvent, heat) improves the sustainability of the fabrication process.

## Graphical abstract

A method to directly produce structurally colored microparticles by drying cholesteric cellulose nanocrystal microdroplets on a superhydrophobic surface is reported. This approach has several key advantages that can unlock large‐scale fabrication of sustainable photonic pigments, including exploiting existing industrial techniques (e.g. aerosolization), reduction in chemicals required (e.g. surfactants), rapid production times (≈40 min or less), and size‐independent and robust color.

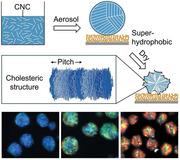

## 1 Introduction

Color is a fundamental form of communication for a huge variety of organisms in nature and can arise from a variety of different mechanisms.^[^ ^1^, ^2^ ^]^ In contrast, within human society coloration is almost exclusively achieved from the incorporation of soluble dyes (commonly molecules) or insoluble pigments (typically dispersed as fine particles) that selectively absorb visible light.^[^ ^3^ ^]^ However, in the last few decades, the range of available visual effects has been expanded through the development of so‐called effect pigments (e.g., precisely‐coated mica particles^[^ ^4^ ^]^), which instead manipulate light by scattering and interference effects. More recently, this concept has expanded toward so‐called photonic pigments, which exploit the selective reflection of visible light from nanostructured microparticles to produce brilliant and long‐lasting structural color.^[^ ^5^, ^6^, ^7^ ^]^

In this context, cellulose nanocrystals (CNCs) have been leading candidates for producing photonic materials that are more sustainable than other commercial counterparts.^[^ ^8^, ^9^, ^10^, ^11^, ^12^, ^13^, ^14^, ^15^, ^16^ ^]^ These elongated nanoparticles can be extracted via acid hydrolysis from natural cellulose sources (e.g., wood pulp, cotton, agricultural waste), and as such are considered an abundant, biocompatible and biodegradable resource.^[^ ^17^ ^]^ In aqueous suspension, colloidally‐stable CNCs are well‐known to spontaneously self‐organize above a critical volume fraction into a cholesteric (i.e., chiral nematic) liquid crystal, whereby the individual nanoparticles locally align along a common direction that spatially rotates to describe a left‐handed helicoid with a defined periodicity (known as the pitch, *p*). This long‐range arrangement can be retained upon water evaporation, which, combined with the intrinsic birefringence of CNCs, results in solid films that display vibrant, iridescent color.^[^ ^12^, ^13^ ^]^

To date, two main strategies have been proposed for the fabrication of photonic pigments from CNCs. A first approach is to produce films, followed by mechanical grinding or ultrasonication into micron‐sized particles.^[^ ^11^, ^18^, ^19^, ^20^ ^]^ However, due to the resultant flake‐like morphology, these CNC pigments exhibit strong angle‐dependent color. (i.e., iridescence). In pursuit of more angle‐independent color, a second approach that is based on emulsification of a CNC suspension followed by water removal has been proposed, enabling direct production of either micron‐sized buckled particles^[^ ^21^, ^22^ ^]^ or spherical shells.^[^ ^23^ ^]^ However, due to the constraints of cholesteric self‐assembly within a spherical geometry, the former can suffer from strong redshifts in color (due to insufficient radial compression of the helicoidal architecture), while the latter requires complex double emulsion droplets and are inherently limited to thin dimensions (and thus weak coloration). Moreover, while droplet production can be upscaled using, for example, membrane emulsification,^[^ ^24^ ^]^ the presence of an encapsulating oil phase usually significantly impedes water loss from the droplets, and thus the drying time remains the key limiting step in CNC pigment production.

Recently, super‐liquid‐repellent surfaces were shown to enable the solvent‐free fabrication of microparticles from microdroplets.^[^ ^25^, ^26^, ^27^, ^28^, ^29^, ^30^ ^]^ Building on this concept, here we demonstrate how drying an aerosolized CNC suspension on such a superhydrophobic surface leads to the direct production of CNC pigments. We observed that structurally colored microparticles achieved via this method have morphological and optical properties similar to those reported for emulsion‐based techniques. Moreover, the air‐dried pigments show stable coloration without the need for solvent or heat post‐treatments, which unlocks methods to tune the color via the initial CNC formulation (e.g., electrolytes, ultrasonication, additives). Finally, by evaporating in a low humidity environment, the fabrication time of the CNC pigments can be reduced to 40 min, without loss of optical performance. This dramatic reduction removes the bottleneck from the upscaling of CNC pigments, which makes this strategy highly appealing for commercial production.

## 2 Results

**Figure 1:**
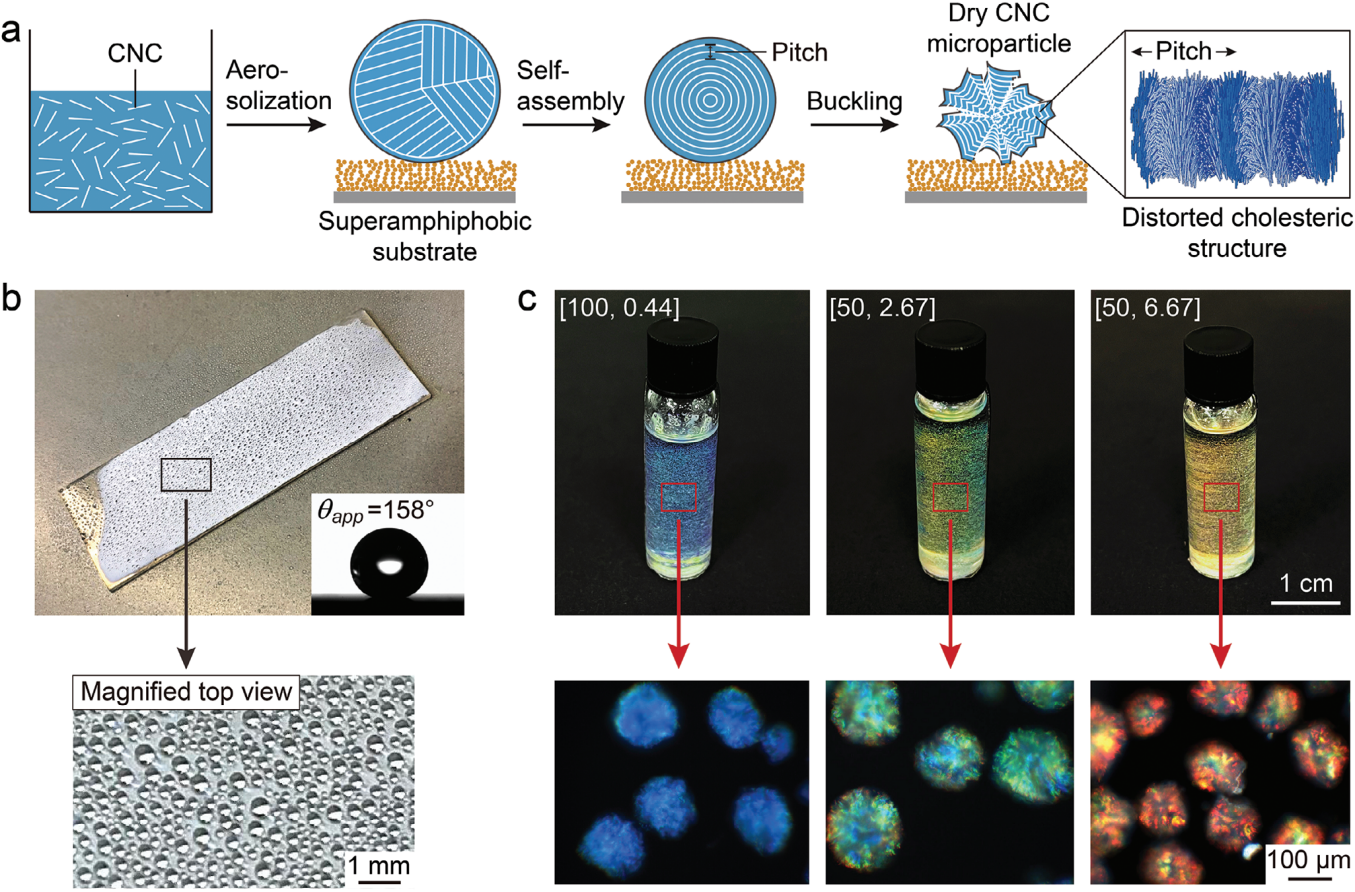
Drying aerosolized CNC microdroplets on a superamphiphobic surface. a) Schematic of the self‐assembly process within an aerosolized CNC microdroplet as it dries on a superamphiphobic surface. The rightmost image shows an inset of a cholesteric CNC structure, indicating the pitch. b) Photograph of the CNC microdroplets on the superamphiphobic surface. The bottom image shows the CNC microdroplets at higher magnification. The inset in the top image shows the high contact angle for a drop of CNC suspension ([CNC]  =  7.0 wt.%) on the superamphiphobic surface. c) Photographs of vials containing photonic CNC pigments suspended in ethyl cinnamate (*n * =  1.56) with blue ([NaCl]/[CNC]  =  100 µmol g^−1^, TS  =  0.44 s mL^−1^), green ([NaCl]/[CNC]  =  50 µmol g^−1^, TS  =  2.67 s mL^−1^), and red coloration ([NaCl]/[CNC]  =  50 µmol g^−1^, TS  =  6.67 s mL^−1^). The bottom insets are unpolarized dark‐field microscopy images in reflection of corresponding CNC microparticles in Cargille refractive index oil (*n*  =  1.57).

To produce photonic microparticles in a single process (**Figure** **1**a), an aqueous CNC suspension was aerosolized into microdroplets, collected onto a substrate, and left to evaporate at room temperature. To ensure that discrete CNC microparticles would be produced after drying and to prevent adhesion it was necessary to avoid wetting of the surface by the aerosolized droplets. To achieve this, a superamphiphobic coating with both superhydrophobic and superoleophobic properties was first deposited onto the glass substrate using the previously reported “soot deposition” method.^[^ ^25^ ^]^ The high apparent contact angle for 5 µL drops of water (θ_*app*_  =  158 ± 1°) and hexadecane (θ_*app*_  =  154 ± 1°), combined with very low drop roll‐off angles (respectively 1 ± 1° and 5 ± 1° for a 10 µL drop) confirmed that this surface has an excellent repellency for both water and organic liquids. As such, the collected CNC microdroplets are expected to be in a non‐wetting “Cassie‐Baxter” state on the superamphiphobic surface (Figure S1, Supporting Information),^[^ ^31^, ^32^ ^]^ whereby the drops only make contact with the nano‐scale tips of this rough morphological surface (Figure S1b, Supporting Information). This ultra‐small liquid‐solid contact area not only guarantees minimal adhesion to the substrate, but crucially allows for a radially‐symmetric drying profile to be achieved within the near‐spherical droplets, analogous to that experienced by emulsified^[^ ^22^ ^]^ or levitated CNC drops.^[^ ^33^ ^]^

The size of the droplets is primarily determined by the parameters of the airbrush and the spraying duration, with longer times resulting in larger droplets (Figure S2, Supporting Information). As an example, spraying for 20 s typically produced CNC microdroplets with a diameter of 240 ± 26 µm (Figure S2, Supporting Information), although the occurrence of larger droplets due to extensive coalescence could not be entirely avoided. It is important to note that the radius of the CNC droplets generated via this method are an order of magnitude or more below the capillary length for water in air (λ_*c*_  =  2.7 mm), thus avoiding gravity‐induced shape deformation.^[^ ^34^ ^]^

To control the drying process, the superamphiphobic substrate laden with freshly deposited CNC microdroplets was immediately placed within an environmental chamber. By maintaining a specific relative humidity within this enclosed chamber, the evaporation rate of the microdroplets could be adjusted to allow for cholesteric self‐assembly to occur. Once dry, the CNC microparticles could then be recovered by either: (i) tilting the substrate such that the microparticles are removed by gravity to yield a dry powder) or (ii) collected by rolling a drop of an apolar liquid across the superamphiphobic surface, which results in a liquid dispersion of colored microparticles (Figure S3, Supporting Information). The choice of an apolar collection liquid avoids redispersal of the individual CNCs that constitute the microparticles, however, based on previous work, we believe that it is possible to use water or any other polar solvent as the collection liquid if heat‐treatment is applied to the microparticles after the evaporation step.^[^ ^21^ ^]^ Figure 1c shows that by varying the initial CNC formulation, this process can be used to produce red, green or blue photonic pigments.

**Figure 2:**
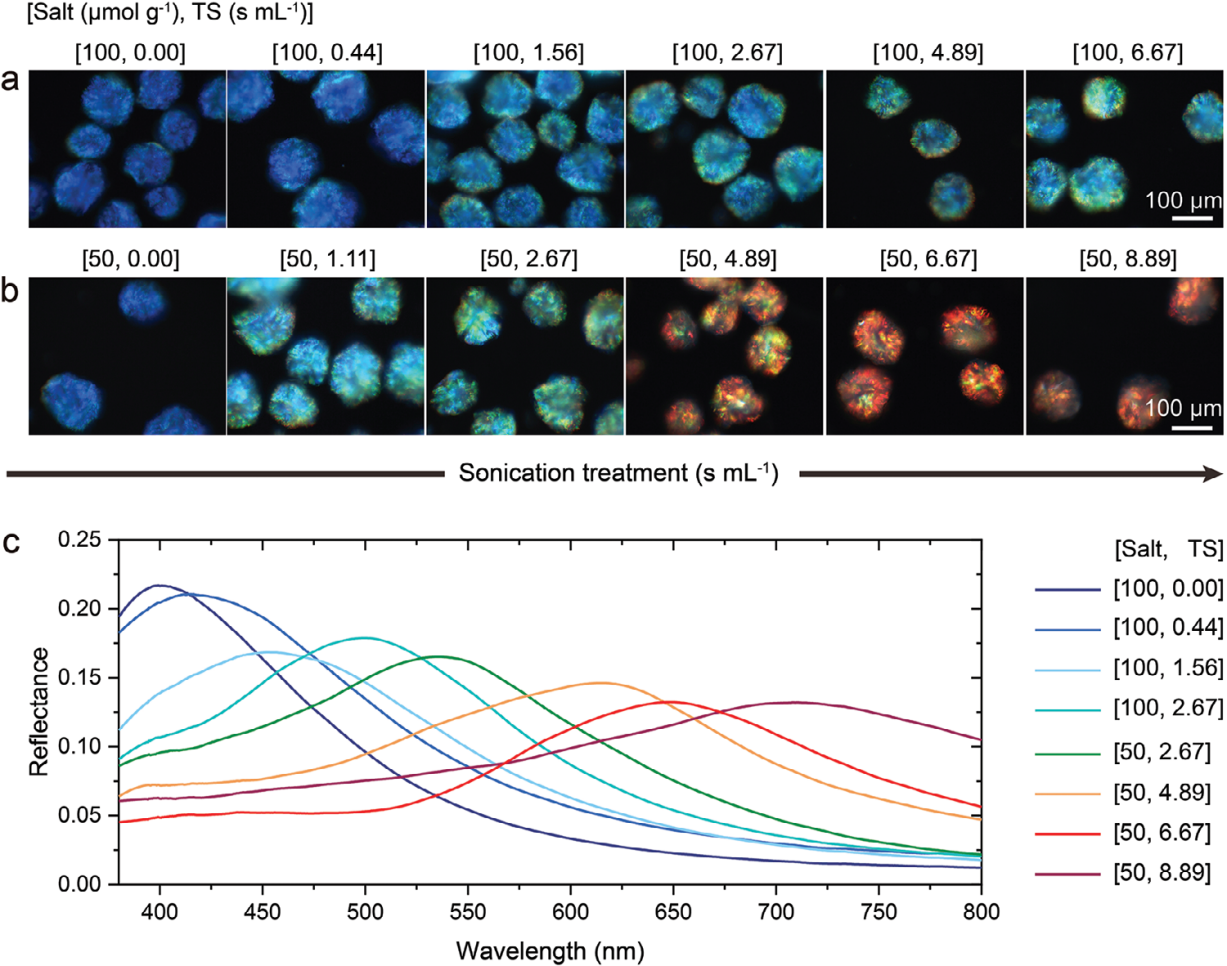
Tuning the color of photonic CNC pigments. Unpolarized dark‐field microscopy images in reflection of CNC microparticles dispersed in an index‐matching oil (*n*  =  1.57) for two different salt concentrations and with increasing ultrasonication dose: a) [NaCl]/[CNC]  =  100 µmol g^−1^ and TS  =  0‐6.67 s mL^−1^, and b) [NaCl]/[CNC]  =  50 µmol g^−1^ and TS  =  0–8.89 s mL^−1^. c) Corresponding unpolarized micro‐spectra averaged over a minimum of 5 microparticles and normalized against a white Lambertian diffuser coated with the same refractive index oil.

To benchmark this approach against our previously reported emulsion‐based method,^[^ ^21^ ^]^ we first attempted to dry the same standard CNC formulation as used in that study. As such, a 7.0 wt.% CNC suspension (University of Maine, pH neutralized with sodium hydroxide) in the presence of additional sodium chloride salt ([NaCl]/[CNC]  =  100 µmol g^[−1]^) was prepared and left to phase‐separate over several days. The lower anisotropic phase was then isolated and used as the formulation for aerosolization onto the superamphiphobic surface, followed by drying in a near‐saturated humidity atmosphere. This process was found to directly produce blue microparticles (λ_max _ =  399 nm, see **Figures** **2**a and S4, Supporting Information). Furthermore, the color of these microparticles was stable, with no change in appearance after exposure to elevated temperatures or polar solvents (Figure 4a) and after long‐term storage in the dark at room temperature for 120 days (Figure S5, Supporting Information). This appearance contrasts strongly against equivalent microparticles produced via the emulsion method,^[^ ^21^ ^]^ which are red upon drying under oil at ambient conditions but can evolve to achieve a similar blue coloration upon subsequent dehydration with a polar solvent (e.g., ethanol, methanol) or by intense heating (e.g., 200 °C for 30 min).

These observations can be explained by considering that the optical properties of the CNC microparticles are fundamentally influenced by the evolution of the cholesteric pitch with increasing CNC volume fraction and, upon reaching the point of kinetic arrest, its geometric compression upon further water loss. In the previous emulsion‐based approach, the observed color was tuned by controlling the amount of residual water trapped within the final microparticle. Removal of this water via post‐treatments resulted in further buckling, leading to a reduction of the cholesteric pitch from *p* ≈ 400 nm to 270 nm, and thus a strong blueshift of the observed color. In contrast, evaporating the CNC microdroplets directly in air ensures the near‐complete removal of water during the evaporation step (as discussed in detail below), leading directly to the maximal amount of buckling for a given CNC suspension. Adjustment of the final color of the microparticles can then be addressed by translating other known methods to tune the cholesteric pitch in dish‐cast films to the microdroplet geometry.

One approach is ultrasonication, which has been shown to break up laterally bound composite particles (i.e., “bundles”) within a CNC suspension.^[^ ^35^ ^]^ This results in a reduced chiral strength of its constituents, leading to an increase in the pitch in suspension and ultimately to a redshift in the dry film.^[^ ^11^, ^35^, ^36^ ^]^ To explore whether this can be applied here, five different CNC suspensions with increasing tip sonication dose (TS  =  0.44, 1.56, 2.67, 4.89, 6.67 s mL^−1^) were used to produce microparticles under identical fabrication conditions. As shown in Figure 2a, a redshift with increasing sonication dose was initially observed up to 2.67 s mL^−1^, after which the color stabilized at cyan‐green. To unlock red hues, the salt concentration and sonication dose were adjusted synergistically, with [NaCl]/[CNC]  =  50 µmol g^−1^ and TS > 4.89 s mL^−1^ found to reach larger wavelengths (Figure 2b). Reflectance micro‐spectroscopy of these pigments confirmed that the CNC microparticles can be fine‐tuned to cover the entire visible spectrum by this approach (Figure 2c). Alternatively, the inclusion of non‐volatile additives is also well known to redshift the color of photonic CNC films,^[^ ^12^, ^37^, ^38^ ^]^ which has been explained by a reduction in the geometric compression of the helicoidal architecture upon drying.^[^ ^39^, ^40^, ^41^ ^]^ We found that this method can also be successfully applied to the air‐dried CNC microparticles, with the inclusion of 30% *w*/*w* of glucose found to cause a shift from blue to green coloration for the standard CNC suspension (Figure S6, Supporting Information).

To better understand the role of kinetic arrest when suspensions are evaporated in a hierarchical architecture (i.e., not the standard film geometry), it is important to understand whether the reflected color is solely determined by the initial CNC formulation, or if the dimensions of the microdroplets also play a role in the self‐assembly process. To do so, we varied the spraying duration (at a fixed spray distance) to produce different‐sized microdroplets with a relatively narrow size dispersity. Figure S7 (Supporting Information) exemplifies differently sized populations of microparticles prepared in this way using the same initial CNC suspension. Optical analysis reveals that all the microparticles exhibit the same peak wavelength independent of their size, while the intensity was found to increase proportionally to their diameter. This validates that the precise size control offered by emulsion‐based methods (and microfluidics in particular) is not required to produce monochromatic photonic CNC pigments at scale. This can be explained by considering that once kinetic arrest has occurred, the self‐assembly process leading to the final structure is dominated by the local compression upon water removal, which is independent of the overall particle size.

**Figure 3:**
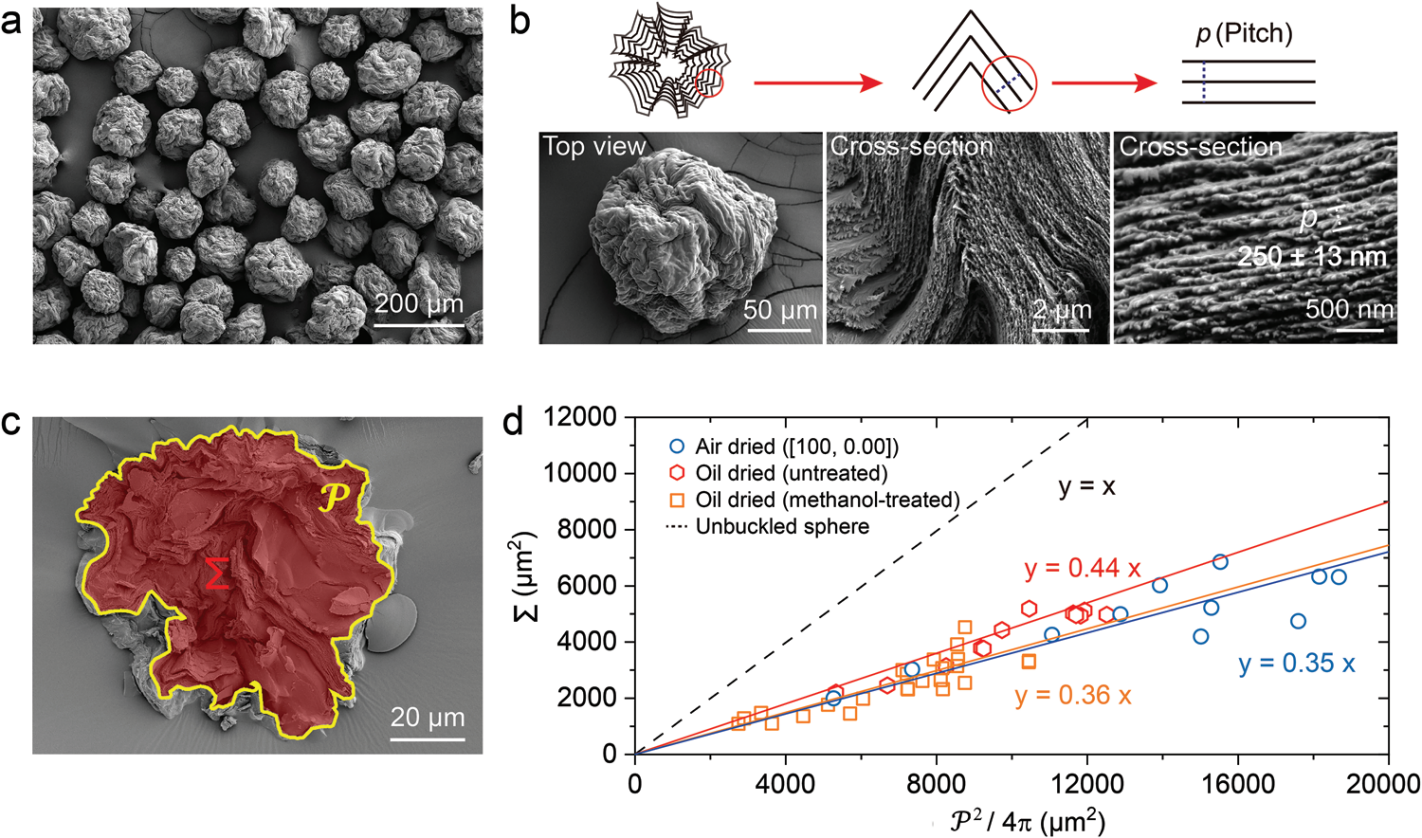
Morphological analysis of the buckled CNC microparticles. a) Top‐view SEM image of microparticles produced from the CNC suspension with [NaCl]/[CNC]  =  100 µmol g^−1^ and TS  =  0 s mL^−1^. b) Corresponding top‐view and cross‐sectional SEM images of a single CNC microparticle, showing the buckled surface morphology and the internal helicoidal structure. c) An example cross‐sectional SEM image of an entire CNC microparticle, with the red area that coincides with the particle cross‐section used to calculate the apparent perimeter (P) and apparent cross‐sectional area (Σ). d) A plot showing the correlation between P and Σ for these CNC microparticles fabricated on superamphiphobic surfaces (blue circles and fitting line). This is compared to previously reported data for microparticles prepared from the same formulation but dried via the emulsion‐based method (red circles and fitting line). The effect of a methanol post‐treatment on these oil‐dried microparticles is also indicated (orange circles and fitting line) respectively.^[^ ^21^ ^]^ The gradient of the fitted line corresponds to the average isoperimetric quotient of the particle cross‐sections (*Q)*.

In stark contrast to flake‐like CNC pigments,^[^ ^18^ ^]^ microparticles produced by the method reported here equally reflect both left‐circular polarized (LCP) and right‐circular polarized (RCP) light (see Figure S8, Supporting Information). This has been previously attributed to radial buckling upon drying, which leads to (i) distortion of the helicoidal architecture (causing elliptically polarized reflection), and (ii) highly tilted cholesteric regions that effectively act as birefringent retardation plates (enabling the interconversion of incident RCP into LCP light and vice‐versa).^[^ ^21^ ^]^ Note that this mechanism is also consistent with the smallest microparticles showing a bias toward LCP reflection (Figure S7, Supporting Information). Scanning electron microscopy (SEM) of red, green and blue CNC microparticles confirmed that they indeed had a highly buckled surface (Figure S9, Supporting Information). Significantly, the degree of buckling is qualitatively similar for microparticles produced from all three formulations, which is consistent with the assertion that complete water loss is achieved when dried in air. Moreover, cross‐sectional SEM of these microparticles confirmed that, despite similar degrees of buckling, the pitch varies in good agreement with the measured optical reflection peaks (**Figure** **3**a,b; Figure S10, Supporting Information). These observations further support the hypothesis that the color tuning of air‐dried CNC microdroplets is driven by differences in cholesteric self‐assembly, rather than by variations in the dehydration‐dependent geometric compression of the microparticles occurring post‐kinetic arrest.

To quantify the degree of buckling, we calculated the isoperimetric quotient of the particle cross‐sections, $Q=4\pi \Sigma /P^{2}$, which can be considered as the ratio of the area of a closed curve Σ to the area of a disk with the same perimeter P (thus the cross‐section of an unbuckled sphere is a perfect circular disk with *Q*  =  1). Using the cross‐sectional SEM images of the blue microparticles produced from the standard CNC suspension, the apparent cross‐section area of the microparticles (Σ) and the corresponding apparent cross‐section perimeter (P) were estimated (Figure 3c). This gives a value of *Q* of 0.35 ± 0.02, which is notably lower than the value reported for equivalent emulsion‐derived CNC microparticles dried in oil (*Q*  =  0.44 ± 0.01) (Figure 3d), but in excellent agreement to the value measured for the same emulsion‐derived microparticles after near‐complete dehydration with methanol (*Q*  =  0.36 ± 0.01).^[^ ^21^ ^]^ This trend is also consistent with the apparent change in diameter observed by optical microscopy upon drying the CNC microdroplets (Figure S11, Supporting Information). Specifically, these measurements revealed that the ratio of the final to initial diameter for microdroplets dried on the superamphiphobic surface reached approximately 0.50, which is again lower than for analogous microparticles prepared by the emulsion‐based method (reported to be approximately 0.57, but decreased to 0.50 after dehydration with methanol).^[^ ^21^ ^]^

**Figure 4:**
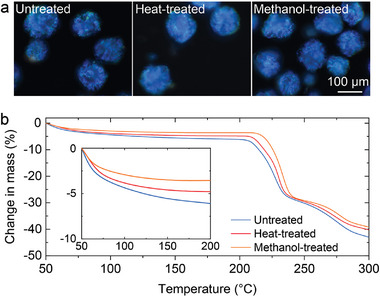
Quantifying the residual water content in the CNC microparticles with and without post‐treatment. a) Unpolarized dark‐field microscopy images in reflection of untreated CNC microparticles prepared on the superamphiphobic surface, as well as after heat post‐treatment (160 °C for 30 min) and methanol post‐treatment (immersion for 5 min). b) Thermogravimetric analysis (TGA) of the untreated, heat‐treated and methanol‐treated CNC microparticles exemplified in (a). The inset shows a magnified view of the mass loss below 200 °C.

To further confirm the greater degree of water loss from the CNC microparticles upon drying in air, thermogravimetric analysis (TGA) was performed to compare the mass loss of untreated, heat‐treated and methanol‐treated pigments upon heating to 300 °C (**Figure** **4**). Below the cellulose degradation point (at around 210–220 °C),^[^ ^42^, ^43^ ^]^ the three samples showed similar traces, with a mass loss of 7.4% attributed to residual water for the air‐dried sample, which decreased slightly to 3.6% after an additional heat treatment at 160 °C for 30 min. This mass loss is again significantly less than that observed for microparticles produced via the emulsion‐based method (35%), but comparable to those that had then undergone subsequent dehydration with methanol (10%).^[^ ^21^ ^]^ Furthermore, air‐dried red CNC pigments show a similar trend (Figure S12, Supporting Information), confirming that the color tuning achieved in this study is not due to differences in water content within the microparticles.

To further rationalize the different drying behaviors between a droplet of CNC suspension suspended in air and emulsified in oil, we consider that the air‐liquid interface surface tension σ is expected to be several times stronger than liquid‐liquid interfacial tension. According to Antonoff's (empirical) rule,^[^ ^44^ ^]^ σ_*w*, *o*_ ≈ σ_*w*_ − σ_*o*_, and given σ_*w*_  =  70 mN m^−1^ in pure water and σ_*o*_ ≈ 25 mN m^−1^ in typical oils, the interfacial tension of a water‐oil interface σ_*w*, *o*_ is expected to be only 35% lower than σ_*w*_.^[^ ^45^ ^]^ However, the presence of surfactant molecules (e.g., 2 wt% Span80) in the oil‐dried droplets is expected to further reduce the water‐oil interface tension to around 4 mN m^−1^,^[^ ^46^ ^]^ which is more than an order of magnitude less than the overall surface tension of these droplets in air. Thus, the much higher surface tension in the air‐dried CNC microdroplet results in a much larger capillary pressure (see Discussion and Figure S13, Supporting Information),^[^ ^45^, ^47^, ^48^, ^49^ ^]^ which is sufficient to overcome the increasing mechanical resistance of the solidifying cholesteric CNC structure to deformation upon drying (when aligned into a Frank‐Pryce‐like configuration, see Panel 3 in Figure 1a),^[^ ^21^, ^50^, ^51^ ^]^ allowing for near‐complete removal of water. Moreover, this increased water loss imposes further contraction between the kinetically arrested CNCs, resulting in smaller, more buckled microparticles and with a proportionally greater reduction in the cholesteric pitch.

Besides the cost of CNC production itself, one of the most significant bottlenecks for the upscaling of photonic CNC materials is the drying time, which often necessitates the use of batch production rather than a more desirable continuous manufacturing process. While many strategies exist to evaporate water quickly (e.g., high airflow, low humidity or elevated temperature), the self‐organization of CNCs into a cholesteric monodomain is driven by their dynamic response to changes in concentration and thus is usually expected to be the limiting process. If the CNC suspension cannot respond quickly enough to the geometric constraints associated with the increasing concentration during this process, the final pitch may be larger and irregular, and the domains may remain small and misaligned, leading to a less vibrant and redshifted appearance. As such, while avoiding the presence of an encapsulating oil layer allows for the CNC microdroplets to be dried much faster in principle, it is necessary to understand what limits cholesteric self‐assembly puts on the maximum evaporation rate.

To investigate the impact of the drying process on the visual appearance of the CNC microparticles, we evaporated microdroplets in different relative humidities. This was achieved using a series of saturated salt solutions (pure water, NaCl, MgCl_2_, LiCl), which are known to equilibrate to a given relative humidity (RH) at a given temperature (respectively: RH  =  100%, 76%, 34%, 12% at 20 °C, Figure S14, Supporting Information).^[^ ^52^ ^]^ In general, it was observed that a lower relative humidity correlates with an accelerated evaporation rate, allowing for the drying time to be significantly reduced (Figure S15, Supporting Information). To quantify the typical drying time, the droplet diameter (*d*) was monitored over time (Figure S15, Supporting Information), and the corresponding surface area (π*d* ^2^) over time was calculated (Figure S16, Supporting Information). From this two quantities were defined: the final drying time *t_d_*, which corresponds to the time when the surface area stopped evolving completely, and the idealized evaporation time *t*_ev_, which is extrapolated from the linear regime of the decrease of the droplet surface area *s*  =  π*d* ^2^(*t*) and corresponds to the evaporation time of a pure water droplet of the same initial size (Figure S16, Supporting Information). The final drying time *t_d_* was found to decrease from 27 h with pure water to 20 h, 2 h and 40 min with saturated NaCl, MgCl_2_ and LiCl solutions, respectively (Figure S17a, Supporting Information). Similarly, the idealized evaporation time *t*_ev_ was also found to decrease (Figure S17b, Supporting Information). The extracted *t*_ev_ allowed for the estimation of the Peclet number, *Pe*, associated with the drying kinetics of these droplets that quantifies the relative effects of advection over diffusion (Figures S17c and S18, Supporting Information).

**Figure 5:**
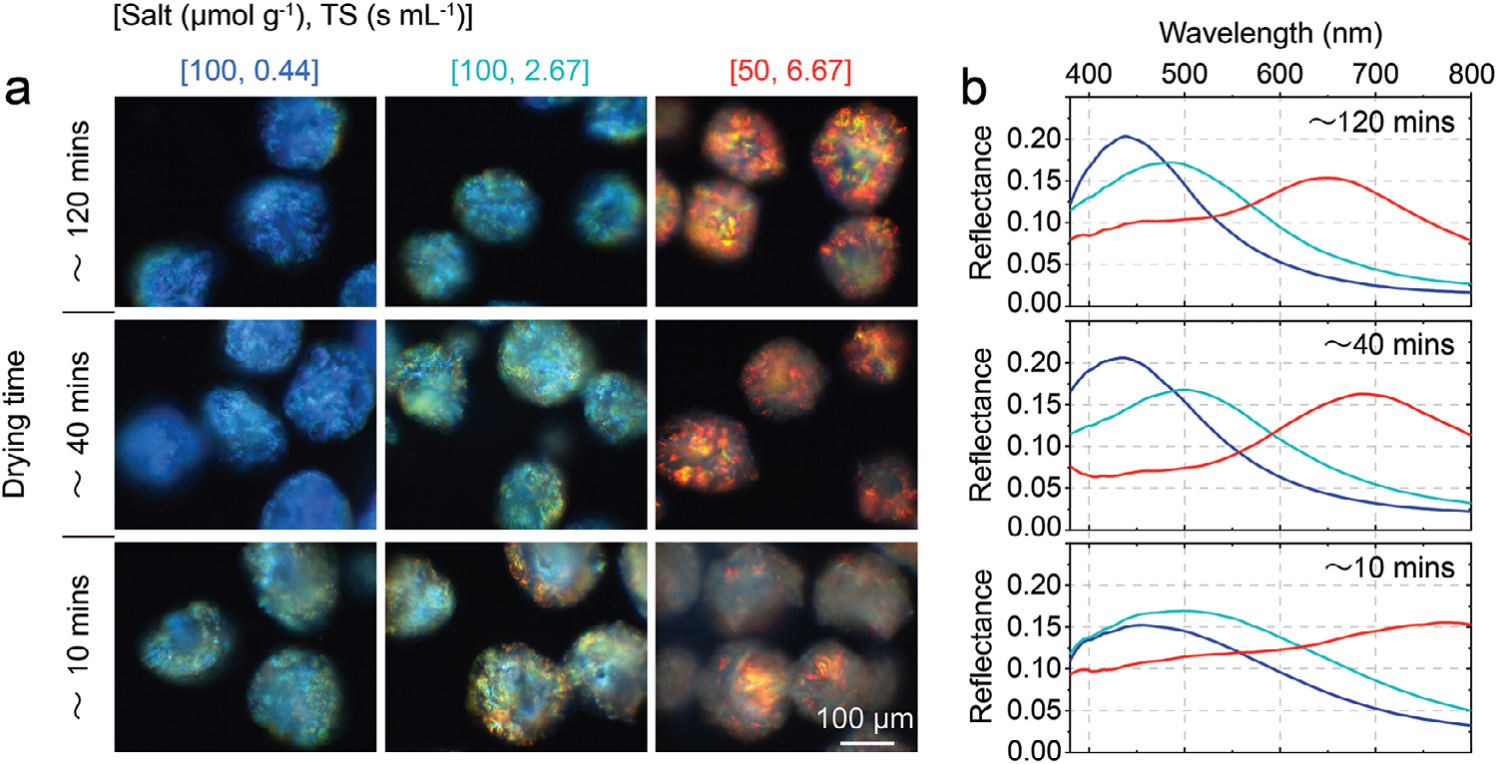
Effect of drying time on the optical properties of CNC pigments. a) Unpolarized dark‐field microscopy images in reflection of representative blue, cyan‐green and red CNC microparticles dried at RH  =  34% (drying time: ≈ 120 min), RH  =  12% (drying time: ≈ 40 min) and under mild vacuum (drying time: ≈ 10 min). b) Corresponding unpolarized micro‐spectra averaged over a minimum of 5 microparticles and normalized against a white Lambertian diffuser coated with the same refractive index oil.

Importantly, it was found that the visual appearance of the red, cyan‐green, and blue CNC pigments produced in these environments were identical (**Figure** **5**; Figure S19, Supporting Information). This greatly reduced drying time can facilitate upscaling of photonic CNC materials. For example, with a drying time of 40 min, the theoretical yield that can be produced on the superamphiphobic surface can be estimated as 0.1 kg m^−2^ d^−1^. The upscaling potential of this method was further evidenced by producing approximately 1 g of CNC photonic pigments (Figure S20, Supporting Information).

While a drying time of 40 min did not compromise the optical quality of the particles, reducing the drying time further to approximately 10 min by applying mild vacuum resulted in an observable change in appearance (Figure 5; Figure S21, Supporting Information). This transition can be attributed to a combination of greater convective flows (that can disrupt long‐range cholesteric order) and an inability to maintain an equilibrium pitch upon water evaporation (leading to uneven color and a redshift in appearance).^[^ ^41^ ^]^ Indeed, the *Pe* associated to each drying microdroplet yielding a uniformly colored particle is *Pe* < 2, except for those dried under vacuum, with a *Pe* ≈ 9. This indicates that the radial advection inside the droplet starts to dominate over the radial diffusion of the CNCs to adjust to the gradient of concentration near the droplet surface.^[^ ^49^ ^]^

To further test the relevance of these pigments for commercial applications, the long‐term color stability of CNC photonic pigments was investigated. An accelerated weathering system was employed to simulate long‐term environmental exposure of CNC pigments to sunlight. In practice, the CNC pigments were fixed onto glass slides and embedded in a poly(dimethylsiloxane) (PDMS) cured matrix. They were then imaged under optical microscopy in dark field reflection mode before the weathering test to provide a control. The samples were then exposed to 100 h of broadband UV light at 50 °C, which corresponds to a blue wool rating of 6, and then imaged again in the same conditions under the optical microscopy. As shown in Figure S22 (Supporting Information), the color of the CNC pigments was still intense after this UV aging, demonstrating their good long‐term color stability. A very mild redshift of color after aging was noticed, which may be due to decomposition products of PDMS under UV illumination that could have diffused into the CNC pigments and resulted in an increase of the pitch of the cholesteric structures.^[^ ^53^ ^]^

While the ability to rapidly dry droplets without disrupting cholesteric self‐assembly removes a major bottleneck in the upscaling of pigment CNC production, the need for an exotic substrate may raise questions regarding the ability to translate this approach to an industrial setting. To address this, we briefly investigated the robustness and scalability of the superamphiphobic coating. As shown in Figure S23 (Supporting Information), contact angle measurements for both water and hexadecane drops on the superamphiphobic substrate before and after 30 cycles of pigment production were found to be consistent. Furthermore, SEM imaging of this surface showed the “micro‐nano” fractal structure remained intact after this repeated usage (Figure S24, Supporting Information). CNC microparticles prepared on superamphiphobic surfaces after this repeated usage were also analyzed by energy‐dispersive X‐ray spectroscopy (EDS), and no fluorine residue was found, which shows there was no release of fluorosilanes from the substrate into CNC microparticles (Figure S25, Supporting Information). Then, to demonstrate the scalability of this approach to produce photonic CNC pigments, we produced much larger superamphiphobic substrates (up to 20 × 30 cm) via the same soot‐deposition method. Six of these larger substrates were combined within a much larger environmental chamber to greatly increase the yield of CNC microparticles to the gram level in each production cycle (Figure S26, Supporting Information).

To illustrate the visual quality of coatings containing CNC pigments, blue, cyan‐green, and red CNC pigments were respectively mixed with polydimethylsiloxane (PDMS) to cover an area of 1.44 cm^2^ on glass slides. Considering there is weak scattering at the buckled microparticle–PDMS interface because of the slight difference in refractive index between PDMS and cellulose, a small amount of carbon black (<0.1 mg) was also added into the PDMS matrix as a broadband absorber. As shown in the macroscopic photo taken outdoors (Figure S27, Supporting Information), blue, cyan‐green, and red colors can be easily from these samples.

Besides the superamphiphobic surface used throughout this work, a superhydrophobic surface was also made to check whether the preparation method for CNC photonic pigments can be extended to such surfaces. The high apparent contact angle for a 5 µL drop of water (θ_*app*_  =  156 ± 1°) and CNC suspension (θ_*app*_  =  154 ± 1°) confirmed that this surface has a good superhydrophobicity (Figure S28a,b, Supporting Information), but this surface could not repel hexadecane (Figure S28c, Supporting Information). CNC suspensions were sprayed onto this superhydrophobic surface following the same procedures. After drying at RH = 12% (using a saturated LiCl solution), CNC microparticles were obtained. As shown in Figure S28d,e (Supporting Information), the CNC microparticles prepared on this superhydrophobic surface exhibited similar colors and micro‐spectra to those prepared on the superamphiphobic surface. Therefore, the preparation method of CNC photonic pigments is not restricted to superamphiophobic surfaces and can be extended to other superhydrophobic surfaces, which further enhances the commercial feasibility of this approach.

## 3 Conclusion

In summary, we demonstrate that photonic CNC pigments can be rapidly produced by drying aerosolized CNC microdroplets on a superhydrophobic surface. We show that the stronger capillary forces present when drying in air result in complete desiccation of the resultant CNC microparticles, which is in marked contrast to those reported previously via emulsification. The enhanced color stability that this affords crucially allows for the final color to be tuned across the visible spectrum by controlling the cholesteric self‐assembly process prior to kinetic arrest (by altering the formulation via ultrasonication, electrolytes, additives, etc.), rather than by manipulation of the degree of geometric compression after kinetic arrest (via controlled removal of residual water).

This one‐step approach has several key advantages for the large‐scale fabrication of photonic CNC pigments, including exploiting existing industrial techniques (e.g., aerosolization and spray‐deposition), a rapid production time (≈40 min), a significant reduction in reagents and chemicals (e.g., surfactants, organic liquids), size‐independent coloration (i.e., tolerant to droplet dispersity), and direct access to a dry pigment powder. Moreover, while a drying time of 10 min shifted the final hue and saturation of the pigments, it is important to reiterate that it still led to visibly colored samples, suggesting that after suitable calibration of the suspension composition and processing conditions, the production rate could be further increased. While this technique relies on the use of superhydrophobic coatings, which commonly use perfluoroalkyl and polyfluoroalkyl substances (PFAS), raising environmental concerns as eternal pollutants, the rapid development of PFAS‐free alternatives^[^ ^54^, ^55^ ^]^ offers promising routes to combine the environmental benefits of cellulose and the CNC microparticle manufacturing process. Moreover, we believe that our proposed technique can be extended to other structurally colored pigment particles obtained by self‐assembly with different types of colloidal building blocks. In conclusion, this method lends itself to industrial production and appears relevant to the adoption of sustainable and biodegradable pigments across a range of applications.

## 4 Experimental Section

### Preparation of Cellulose Nanocrystal Suspension

The aqueous CNC suspension was purchased from the Process Development Center of the University of Maine (Batch no. 2021‐FPL‐170, pH neutralized with sodium hydroxide, 1.1 wt.% sulfur content as reported by manufacturer) and had a concentration of [CNC]  =  10.44 wt.% (A&D, MX‐50 moisture analyzer). The as‐received CNC suspension was diluted with type 1 ultrapure water (Milli‐Q, Millipore, Synergy UV system) and aqueous sodium chloride solution ([NaCl]  =  0.1 mol L^−1^) to obtain suspensions with a final CNC concentration of 7.0 wt.% and with [NaCl]/[CNC] ratios of either 50 µmol g^−1^ or 100 µmol g^−1^ (Note that in contrast to dish‐cast films,^[^ ^11^ ^]^ salt was required in all formulations to access reflection at visible wavelengths, see Figure S4, Supporting Information). Afterwards, to tune the final color of the microparticles, these suspensions were treated by ultrasonication (Fisherbrand 505 Sonic Dismembrator 500 W, amplitude  =  40%, tip diameter  =  12.7 mm, sample volume  =  45 mL) for a time interval between 20 and 400 s. The tip sonication dose (TS) is reported in units of s mL^−1^, as it was shown to be the most appropriate for tuning CNC properties.^[^ ^35^ ^]^ Finally, the suspensions were left unperturbed for several days to allow for phase separation to occur, as evidenced by a distinct stratification into two layers when viewed between crossed polarizers. Once the relative proportion of these layers stopped evolving further, the bottom anisotropic layer was used for fabricating the CNC microparticles.

### Fabrication of Superamphiphobic Surfaces

A uniform layer of black soot was deposited onto a glass slide by moving it through the flame of a burning candle for ≈3 min. Next, the soot‐coated glass slides were placed in a desiccator alongside a glass vial containing tetraethoxysilane (Sigma‐Aldrich, 4 mL) and a second vial containing aqueous ammonia solution (Sigma‐Aldrich, 4 mL). The Stöber reaction was initiated on the candle soot‐coated glass slide by chemical vapor deposition under mild vacuum in a desiccator over a minimum of 24 h. Subsequently, the treated slides were subjected to incineration at 550 °C for 2 h to remove the candle soot, followed by exposure to an open glass vial containing 1H,1H,2H,2H‐perfluorodecyltrichlorosilane (Sigma‐Aldrich. 300 µL) under mild vacuum in a desiccator for 2 h at ambient temperature.

### Fabrication of Superhydrophobic Surfaces

Commercial Ultra‐ever Dry paint was purchased from Ultra Tech International, Inc. and sprayed on glass slides to form a superhydrophobic coating following the supplier's guidance notes.

### Wetting Characterization

The apparent static water contact angle (θ_*app*_) and roll‐off angle (θ_*roll*_) measurements were performed on an optical contact angle measuring system (Dataphysics OCA 50 AF). To measure θ_*app*_, a 5 µL drop was placed on the sample surface and the at‐rest angle between the tangent to the liquid‐vapor interface and the solid surface was recorded. For measurement of θ_*roll*_, a 10 µL drop was placed on the sample surface and the substrate was tilted at a speed of 0.1° s^−1^. The value of θ_*roll*_ was recorded at the onset of drop rolling.

### Production of Cellulose Nanocrystal Microparticles

The specified CNC suspension was aerosolized onto the superamphiphobic surfaces using an airbrush (Sparmax SP‐35). In order to obtain consistently sized microdroplets, the following parameters were chosen: driving pressure of 0.4 bar, nozzle diameter of 0.35 mm, and a spray distance of ≈18 cm from the substrate. Figure S2 (Supporting Information) displays the relationship between spraying time and CNC droplet diameter, showing a clear size increase with increasing spraying time. At 5, 12, 20, and 30 s spraying times, the average CNC droplet diameters were 63.97 ± 9.77, 175.01 ± 14.94, 239.12 ± 26.27, and 300.27 ± 21.95 µm, respectively. Unless otherwise stated, all microdroplets in this study were produced using a 20 s spray duration. Immediately after spraying, the superamphiphobic substrate (25 mm × 60 mm) loaded with CNC microdroplets and a small dish (diameter × height = 40 mm × 5 mm) containing Milli‐Q water or a saturated salt solution were placed under a larger Petri dish (diameter × height = 90 mm × 7 mm), to isolate the sample from the external environment. As shown in Figure S14 (Supporting Information), the presence of pure water or various saturated salt aqueous solutions enabled control over the relative humidity within the Petri dish, thereby regulating the drying speed of CNC microdroplets. The corresponding relative humidity at 20 °C for pure water, saturated NaCl solution, saturated MgCl_2_ solution and saturated LiCl solution is reported to be 100%, 76%, 34% and 12%, respectively.^[^ ^52^ ^]^

### Optical Characterization

To reduce broadband scattering from the particle‐air interface that arises from the buckled morphology, the CNC microparticles were dispersed in refractive index‐matching oil during microscope analysis (Cargille Refractive Index Liquid Series A, $n_{D}^{25}$ = 1.5700). A customized Zeiss Axioscope A1 microscope with a CMOS camera (Pixelink PL‐D725CU‐T, color balanced with a white diffuser) was used for collecting optical micrographs. A Halogen lamp (ZEISS, HAL100) was used as a light source in Koehler illumination. Dark‐field images were taken through a Zeiss EC Epiplan‐Apochromat objective (×20, NA 0.6), with the microscope configured such that the illumination was limited by the NA of the objective. The reflected light could also be filtered with a quarter‐waveplate and a linear polarizer mounted at different orientations to distinguish between left‐ or right‐handed circularly polarized light. To collect micro‐spectroscopy, the microscope was coupled to a spectrometer (Avantes AvaSpec‐HS2048) using an optical fiber (Avantes FC‐UV200‐2‐SR, 200 µm core size) in confocal configuration. The area used to collect the micro‐spectra was ≈30 µm in diameter and was centred on the particle of interest. The reflectance spectra were normalized in dark‐field against a white diffuser (Labsphere SRS‐99‐010) coated with the same refractive index oil (Figure S29, Supporting Information). The timelapse series of drying CNC microdroplets was recorded on the same microscope in bright field using a Zeiss EC Epiplan‐Neofluar objective (×5, NA 0.13), and the microdroplets was prepared from the bottom anisotropic layer of the CNC suspensions ([NaCl]/[CNC]  =  100 µmol g^−1^, TS  =  0 s mL^−1^, [NaCl]/[CNC]  =  100 µmol g^−1^, TS  =  0.44 s mL^−1^). Photographs of vials containing CNC microparticles dispersed in ethyl cinnamate (Sigma‐Aldrich, $n_{D}^{20}$  =  1.558) were recorded under diffuse illumination (i.e., fluorescent ceiling light) with an Apple iPhone 13 smartphone.

### Characterization by Dynamic Light Scattering (DLS)

The bottom anisotropic layer of the CNC suspension ([NaCl]/[CNC]  =  100 µmol g^−1^, TS  =  0.44 s mL^−1^) was diluted with ultrapure water to obtain a CNC concentration of 0.1%. Additional sodium chloride was added to the suspension to adjust the salt concentration to 1 mM. DLS analysis was then performed on the prepared CNC suspension using a Zetasizer (Nano series, Malvern Panalytical Ltd.) to determine the diffusion coefficient of the CNCs.

### Morphological Characterization by Scanning Electron Microscopy (SEM)

To observe the surface morphology of the CNC microparticles, conductive carbon tape was employed to transfer them onto flat stubs, which were subsequently coated with a 10 nm layer of platinum using a sputter coater (Quorum Q150T ES). To image the interior of the microparticles, they were embedded in cellulose butyrate acetate, followed by freeze‐drying in liquid nitrogen for 5 min and immediate mechanical crushing. The resulting fragments of the microparticle‐containing film were mounted to vertical stubs using the above procedure.

### Thermogravimetric Analysis (TGA)

CNC microparticles were measured using a Q50 TGA instrument, with the following parameters: sample mass  =  10‐15 mg, nitrogen flow  =  100 mL min^−1^, heating rate  =  2 °C min^−1^, temperature range  =  30‐300 °C.

### Long‐Term Color Stability

Blue, cyan‐green, and red CNC microparticles dried at RH = 12% were transferred onto glass slides and covered by uncured PDMS (Sylgard 184 from the Dow Chemical Company), respectively. The weight mixing ratio between the base and the cure agent was 10:1 for PDMS. After being in an oven at 60 °C for ≈ 1 h, PDMS was cured and fixed the position of CNC pigments. Dark‐field images were taken through a Zeiss EC Epiplan‐Apochromat objective (×10, NA 0.3). Then these samples were exposed to an accelerated weathering system (Sevar Bandol Wheel H 400) at 50 °C, using a mercury bulb as the light source. The samples were exposed to 100 h doses, corresponding to a blue wool rating of 6. After aging, dark‐field images were taken again for the same CNC microparticles.

### EDS Analysis

CNC photonic pigments made on the superamphiphobic surface were added onto a conductive carbon tape and then transferred onto a flat stub. 10 nm gold was coated onto the pigments for improved conductivity using a sputter coater (KAIPLE SD‐900). A SEM (Phenom Pro X) with EDS capability was used for the EDS analysis.

### CNC photonic Pigments Embedded in a PDMS Matrix

40 mg of prepared blue, cyan‐green, and red CNC pigments dried at RH = 12% were respectively mixed with 150 mg of uncured polydimethylsiloxane (PDMS) (Sylgard 184 from the Dow Chemical Company) and a small amount of carbon black (<0.1 mg) to cover an area of 1.44 cm^2^ on glass slides. The weight mixing ratio between the base and the cure agent was 10:1 for PDMS. The weight ratio between carbon black and CNC pigments was smaller than 1/400. Carbon black was added into the binder matrix to absorb unwanted scattering at the buckled microparticle–PDMS interface. Then these samples were put into an oven at 60 °C for ≈ 1 h to cure PDMS.

## Conflict of Interest

The authors declare no conflict of interest.

## Supporting information



Supporting Information

## Data Availability

Original data relating to this publication is available from the University of Cambridge data repository (https://doi.org/10.17863/CAM.115407).
