# Supplementary material for: Spray‐Assisted Fabrication of Cellulose Photonic Pigments on Superhydrophobic Surfaces
Source: Adv Mater. 2025 Jan 29;37(22):2416607. doi: 10.1002/adma.202416607 (PMC12138870; doi:10.1002/adma.202416607)
Supplement: Supplementary file 1 — Supporting Information [file ADMA-37-2416607-s001.pdf]

ADVANCED MATERIALS

Supporting Information

for *Adv. Mater.*, DOI 10.1002/adma.202416607

Spray-Assisted Fabrication of Cellulose Photonic Pigments on Superhydrophobic Surfaces

Jianing Song, Richard M. Parker, Bruno Frka-Petesic, Tao Deng, Luqing Xu, Xu Deng, Silvia Vignolini* and Qingchen Shen**

Supplementary Materials

Spray-assisted fabrication of cellulose photonic pigments on superhydrophobic surfaces

Jianing Song[1,2,3], Richard M. Parker[1], Bruno Frka-Petesic[1,4], Tao Deng[5], Luqing Xu[3], Xu Deng[2,3], Silvia Vignolini[1,6]*, and Qingchen Shen[1,6]**

[1.] Yusuf Hamied Department of Chemistry, University of Cambridge, Lensfield Road, CB2 1EW, United Kingdom.

[2.] Shenzhen Institute for Advanced Study, University of Electronic Science and Technology of China, Shenzhen 518110, P. R. China.

[3.] Institute of Fundamental and Frontier Sciences, University of Electronic Science and Technology of China, Chengdu 611731, P. R. China.

[4.] International Institute for Sustainability with Knotted Chiral Meta Matter (WPI-SKCM[2]), 1-3-1 Kagamiyama, Hiroshima University, Higashi-Hiroshima, Hiroshima, 739-8526, Japan.

[5.] State Key Laboratory of Metal Matrix Composites, School of Materials Science and Engineering, Shanghai Jiao Tong University, Shanghai 200240, P. R. China.

[6.] Max Planck Institute of Colloids and Interfaces, Science Park Golm, 14476 Potsdam, Germany.

Supplementary Discussion

Calculation of the capillary pressure

The capillary pressure P_{cap} in the drying droplet is due to the Laplace pressure developing due to the local curvature of the water interface:

$$P_{cap} = \sigma_i \left(\frac{1}{\varrho_1} + \frac{1}{\varrho_2}\right)$$

where σ_i is the surface tension of the interface, namely σ_w = 70 mN/m for water in air and σ_{span80} = 4 mN/m for water-in-oil droplets in the presence of Span80 surfactant.[1] Here, the relevant curvatures (ϱ_1, ϱ_2) acting on the particles are not the radius of the droplets, but the curvatures developing transiently when the individual particles are reluctant to remain in the water phase: an individual particle surrounded by water that would not follow the receding water-oil or water-air interface would cause the interface to bulge and cause a local Laplace pressure scaling with the interparticle distance. More specifically, if we define the Wigner-Seitz (WS) cell of the rods, corresponding to a geometric shape where each individual CNC is surrounded by its specific volume of water, then the description of the Laplace pressure can be explicated, on average, as:

$$P_{cap,3D} = \frac{2\sigma_i}{\varrho_{3D}}$$

with $\varrho_1 = \varrho_2 = \varrho_{3D}$ defined as

$$\varrho_{3D} = \left(\frac{3V_{CNC}}{4\pi\Phi}\right)^{1/3}$$

with $V_{CNC} = L_{CNC} \times W_{CNC} \times T_{CNC}$ the average volume of a CNC of length L_{CNC}, width W_{CNC} and thickness T_{CNC}, and Φ their volume fraction.

If we consider the CNCs as arranged in a locally aligned fashion, alike a hexagonal packing of rods, the pressure would correspond to a curvature only along one dimension ($\varrho_1 = \varrho_{2D}$), scaling with a cylindrical WS cell, while the other direction will have zero curvature ($\varrho_2 = 0$). The corresponding pressure would then scale as:

$$P_{cap,2D} = \frac{\sigma_i}{\varrho_{2D}}$$

with ϱ_{2D} defined as

$$\varrho_{2D} = \left(\frac{W_{CNC}T_{CNC}}{\pi\Phi}\right)^{1/2} = \frac{a}{\sqrt{\Phi}}$$

where an effective CNC cylindrical radius $a = \sqrt{W_{CNC}T_{CNC}/\pi}$ was introduced. For the sake of an estimation of these pressures, we assumed the CNCs have the following average dimensions: $L_{CNC} = 130$ nm, $W_{CNC} = 25$ nm and $T_{CNC} = 7$ nm.

Assuming an initial droplet diameter of 300 µm and an initial concentration of CNCs of c_{CNC} = 7 wt%, we can estimate the capillary pressure as the droplet shrinks over time (see **Figure S13**). From the equations above, the pressure retaining the CNCs in the water phase in the initial droplet gives a similar order of magnitude for the 2D or the 3D models, and ranges between 2 and 3 MPa in air and 0.11 and 0.16 MPa in oil, depending on the chosen model. As the droplet dries and the interparticle distance gets smaller, the pressure further increases, reaching about 8.7 ± 0.7 MPa in air and only 0.50 ± 0.05 MPa in oil (assuming the surface tension is constant and equal to the starting suspension, which is likely to represent an upper limit).

Supplementary Figures

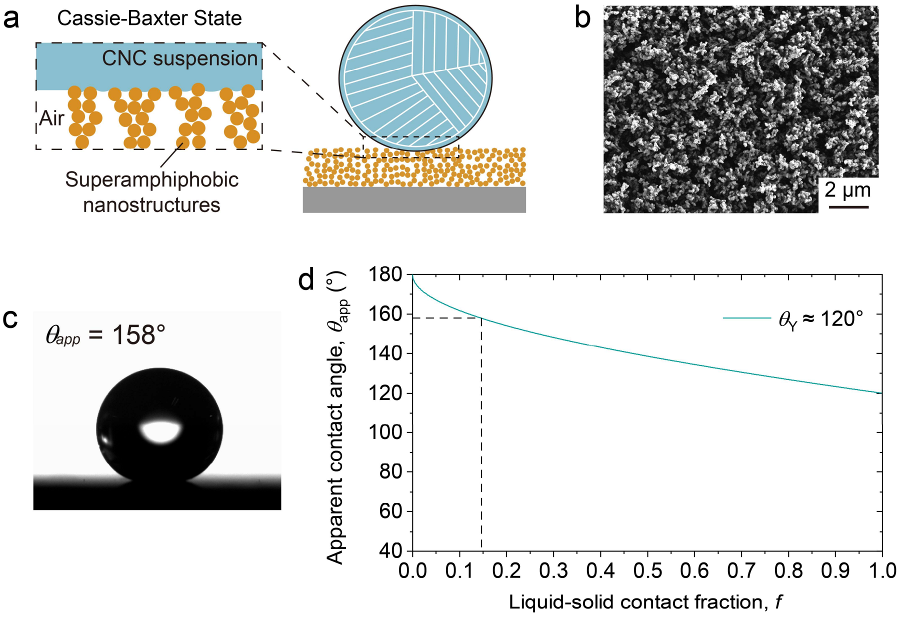

Figure S1. (a) Schematic of a CNC droplet on a superamphiphobic surface. **(b)** Top-view SEM image of the superamphiphobic surface topography. **(c)** Side-view of a 5 µL drop of CNC suspension ([CNC] = 7.0 wt.%) on the superamphiphobic surface highlighting the apparent contact angle θ_{app}. **(d)** Relationship between the apparent contact angle θ_{app} and the liquid-solid contact fraction f for an ideal Cassie-Baxter state for the Young's contact angle θ_Y of water (measured on a flat fluorinated solid surface). Young's equation is expressed as: $\cos\theta_{app} = f(1+\cos\theta_Y) - 1$.[2, 3] The dashed lines indicate the *f* value for the aqueous CNC droplet on the candle-soot-based superamphiphobic surface.

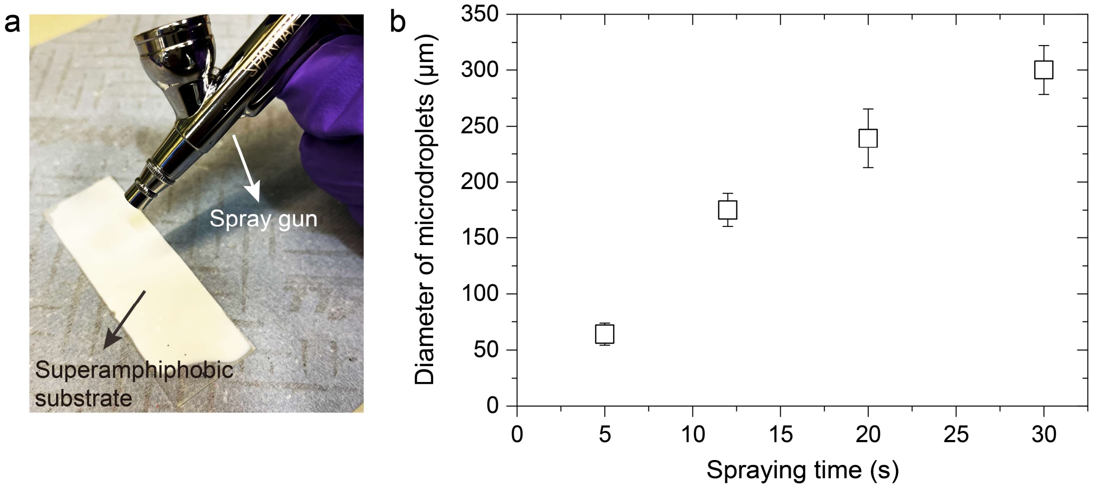

Figure S2. (a) Photograph of the spray gun and superamphiphobic surface for preparing aerosolized CNC microdroplets. **(b)** The relationship between the average diameter of the CNC microdroplets on the superamphiphobic surface and the spraying time for a fixed distance of approx. 18 cm from the substrate. Other spraying parameters are a driving pressure of 0.4 bar and a nozzle diameter of 0.35 mm.

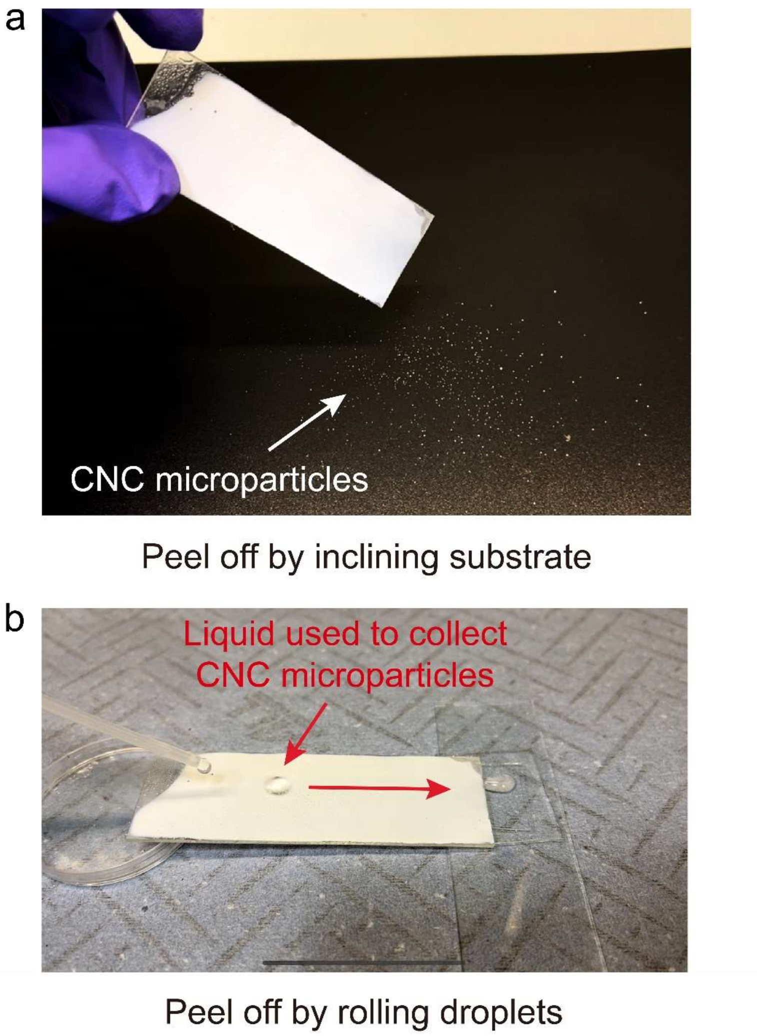

Figure S3. Two methods to recover CNC microparticles from the superamphiphobic substrate were explored: **(a)** tilting the substrate to release the microparticles under gravity in a dry state, and **(b)** accumulation within larger drops of refractive index oil (or other suitable liquid) that are rolled across the superamphiphobic surface. The choice of an apolar collection liquid guarantees no redispersal of individual CNCs, which maintains the integrity of the CNC microparticles and directly yields a liquid dispersion of colored microparticles.

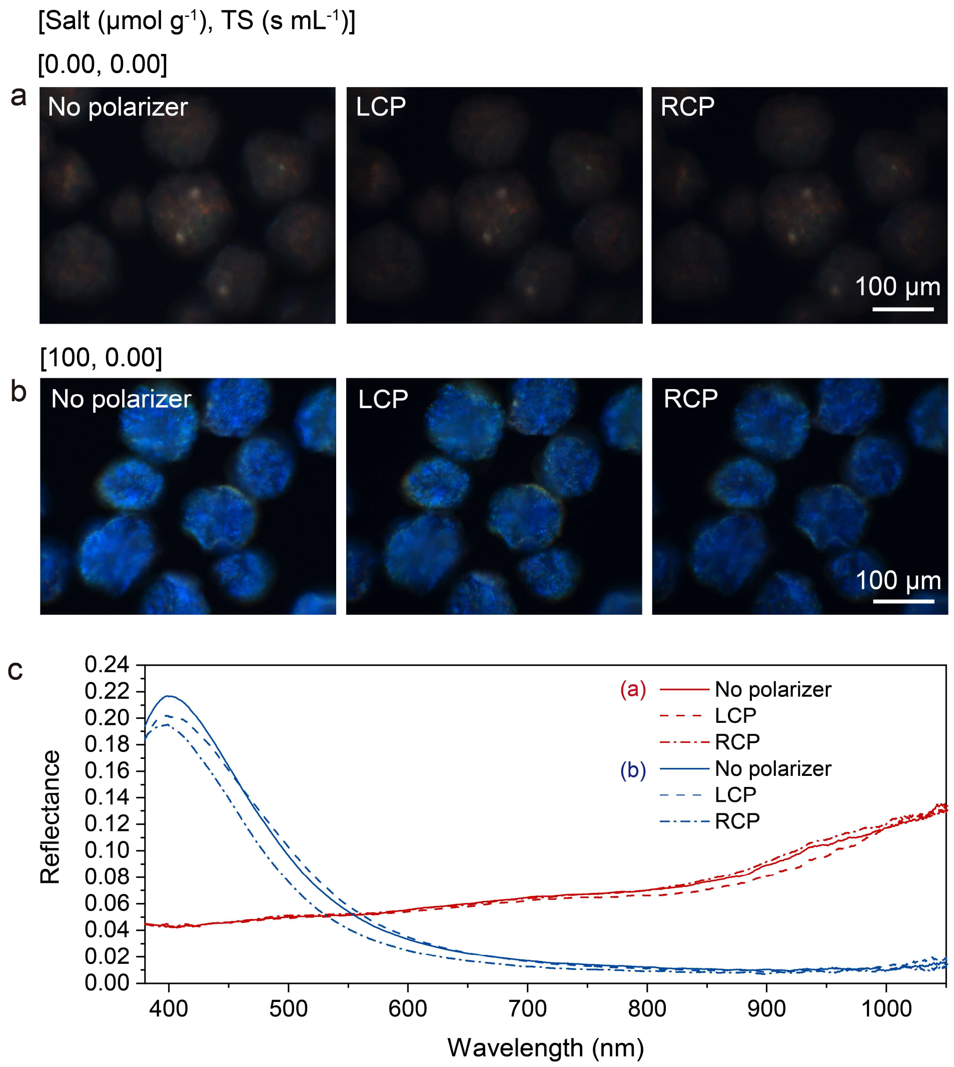

Figure S4. Dark-field microscope images in reflection of CNC microparticles dispersed in an index-matching oil (Cargille Series A, $n = 1.57$) prepared with and without additional salt: **(a)** [NaCl]/[CNC] = 0 μmol g^{-1}, TS = 0 s mL^{-1} and **(b)** [NaCl]/[CNC] = 100 μmol g^{-1}, TS = 0 s mL^{-1}. The images are either unpolarized or analyzed with either an LCP or RCP filter. **(c)** Corresponding micro-spectra averaged over 5+ microparticles and normalized against a white Lambertian diffuser coated with the same refractive index oil.

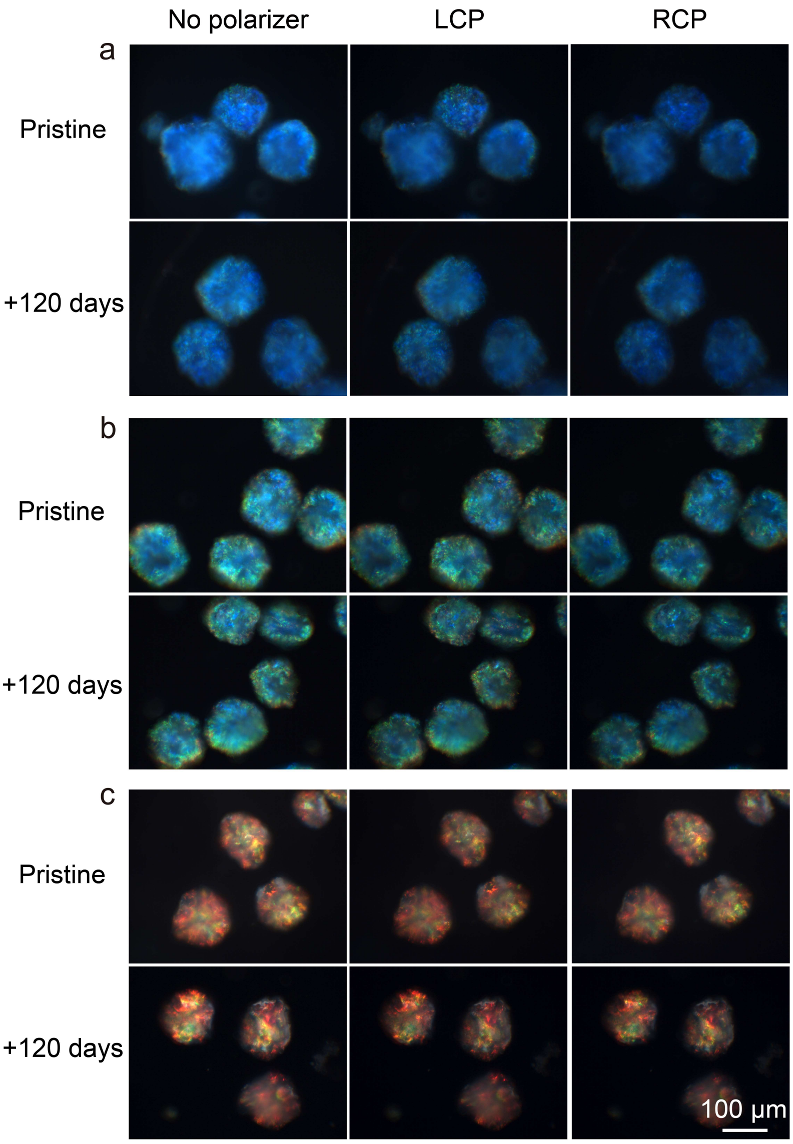

Figure S5. Dark-field microscopy images in reflection of **(a)** blue ([NaCl]/[CNC] = 100 μmol g^{-1}, TS = 0.44 s mL^{-1}), **(b)** cyan-green ([NaCl]/[CNC] = 50 μmol g^{-1}, TS = 2.67 s mL^{-1}) and **(c)** red ([NaCl]/[CNC] = 50 μmol g^{-1}, TS = 6.67 s mL^{-1}) CNC microparticles dried at RH = 100% and the same ones 120 days later (stored in an index-matching oil (n = 1.57) in the dark at room temperature).

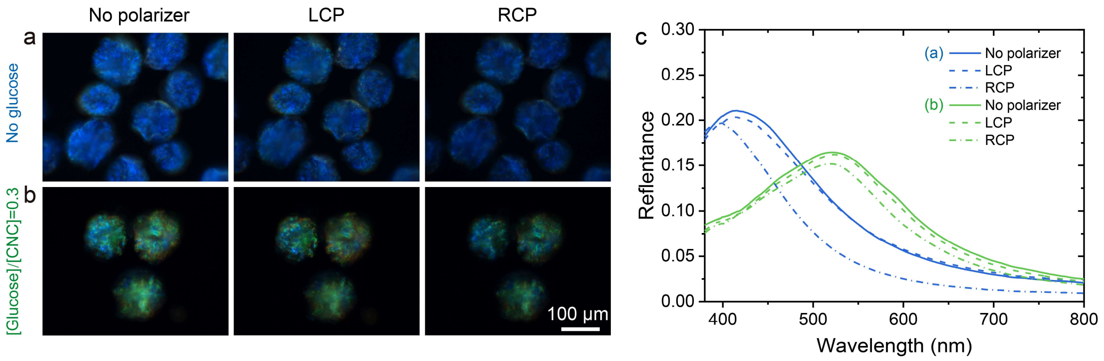

Figure S6. Dark-field microscope images in reflection of CNC microparticles dispersed in an index-matching oil (Cargille Series A, $n = 1.57$) prepared with and without an additive: **(a)** [NaCl]/[CNC] = 100 μmol g^{-1}, TS = 0 s mL^{-1} and **(b)** [NaCl]/[CNC] = 100 μmol g^{-1}, [glucose]/[CNC] = 30 wt.%, TS = 0 s mL^{-1}. The images are either unpolarized or analyzed with either an LCP or RCP filter. **(c)** Corresponding micro-spectra averaged over 5+ microparticles and normalized against a white Lambertian diffuser coated with the same refractive index oil.

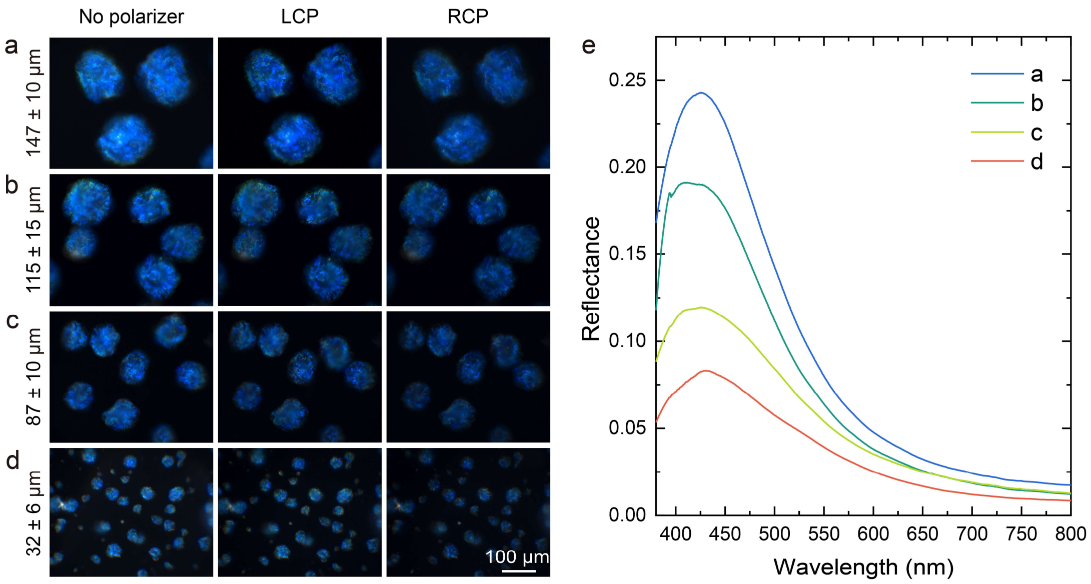

Figure S7. (a-d) Dark-field microscopy images in reflection of CNC microparticles ([NaCl]/[CNC] = 100 µmol g^{-1}, TS = 0.44 s mL^{-1}) with different average diameters, as prepared by varying the spraying duration. The microparticles were dispersed in an index-matching oil (n = 1.57) and imaged either without a filter or through an LCP or RCP filter. **(e)** Micro-spectroscopy of the CNC microparticles exemplified in (a-d), recorded without a filter and averaged over 3+ microparticles.

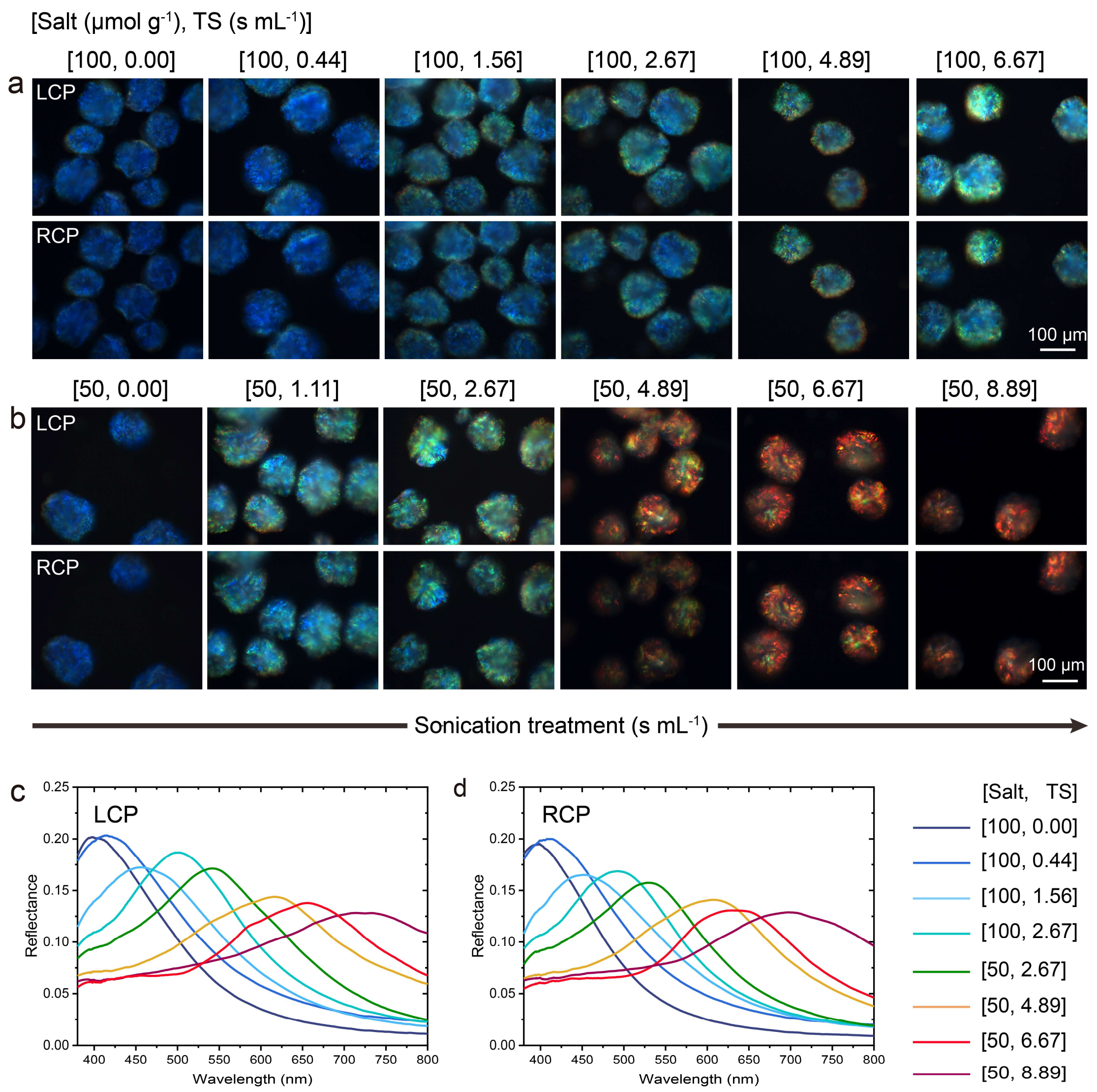

Figure S8. Dark-field microscopy images in reflection of CNC microparticles prepared from two series of CNC suspensions with increasing ultrasonication dose for a fixed salt concentration of **(a)** [NaCl]/[CNC] = 100 μmol g^{-1} and **(b)** [NaCl]/[CNC] = 50 μmol g^{-1}. The microparticles are dispersed in an index-matching oil ($n = 1.57$) and the images are analyzed with either an LCP or RCP filter. **(c)** Corresponding micro-spectra under an LCP filter averaged over 5+ microparticles. **(d)** Corresponding micro-spectra under an RCP filter averaged over 5+ microparticles. The spectra were normalized against a white Lambertian diffuser coated with the same refractive index oil. Corresponding unpolarized images and spectra can be found in Figure 2.

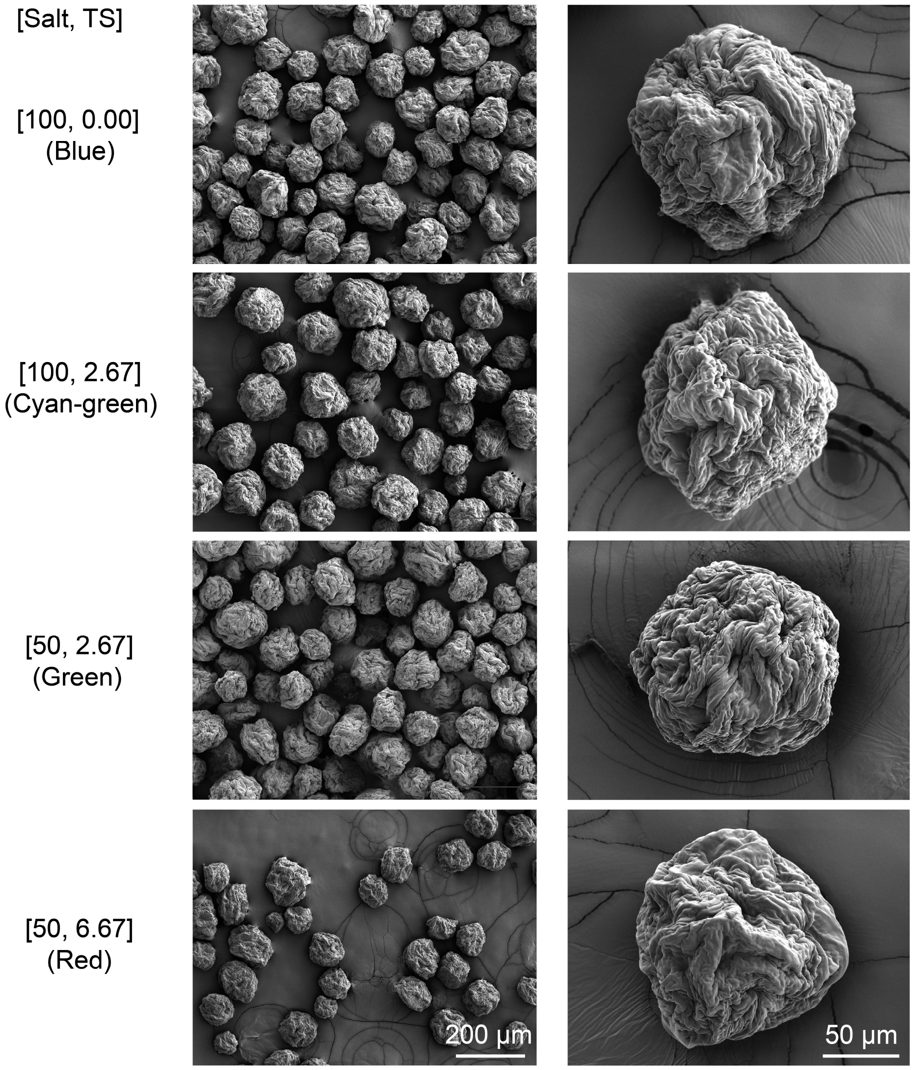

Figure S9. The surface morphology of multiple and single CNC microparticles, as recorded by scanning electron microscopy (SEM): blue pigments ([NaCl]/[CNC] = 100 μmol g^{-1}, TS = 0 s mL^{-1}), cyan-green pigments ([NaCl]/[CNC] = 100 μmol g^{-1}, TS = 2.67 s mL^{-1}), green pigments ([NaCl]/[CNC] = 50 μmol g^{-1}, TS = 2.67 s mL^{-1}), and red pigments ([NaCl]/[CNC] = 50 μmol g^{-1}, TS = 6.67 s mL^{-1}).

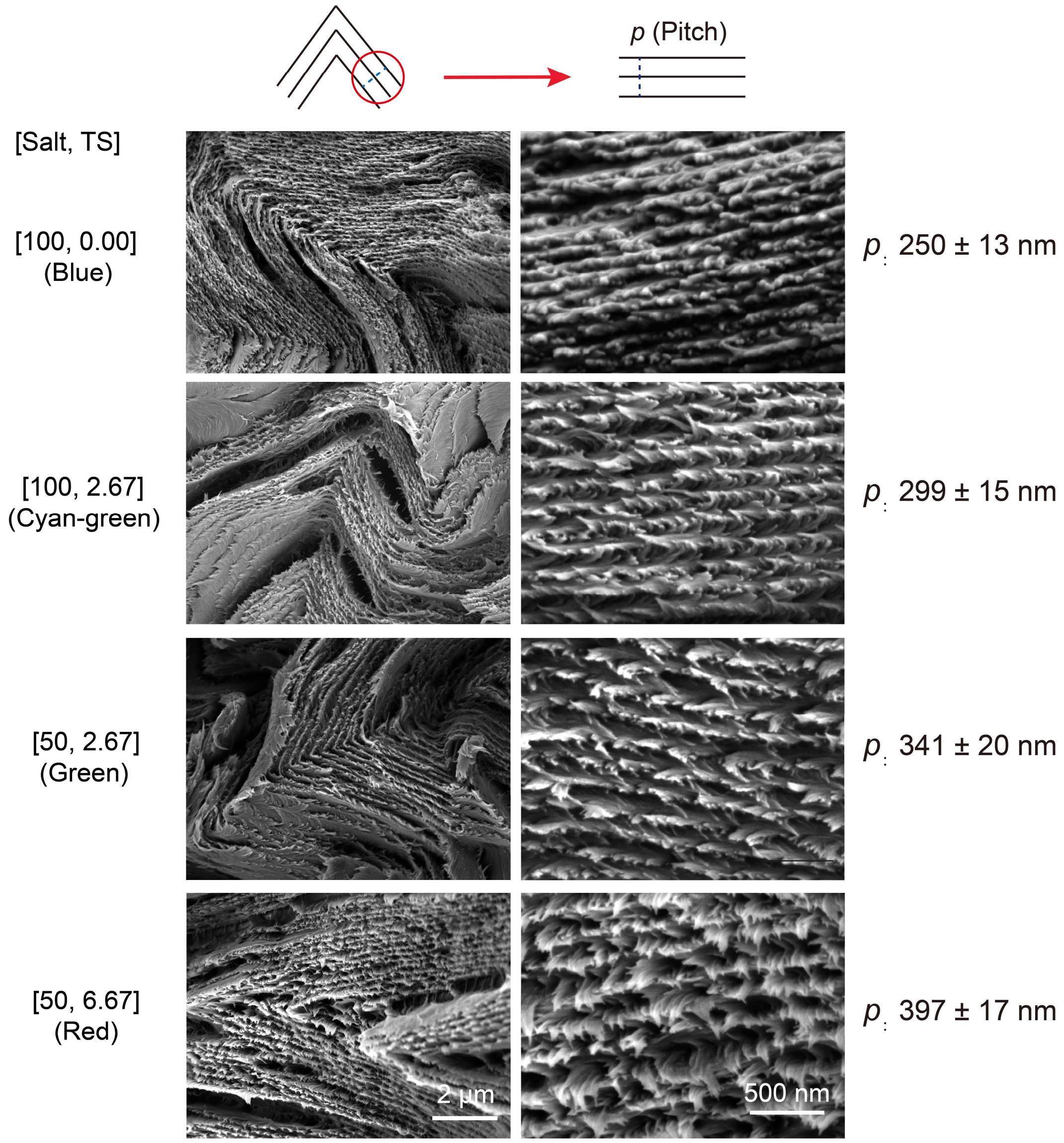

Figure S10. Cross-sectional SEM images of the microparticles in Figure S8, confirming that the pitch *p* of the helicoidal structure increases with the redshift of observed colors. *p* was measured from the limb of a fold, which is primarily responsible for the visual appearance.

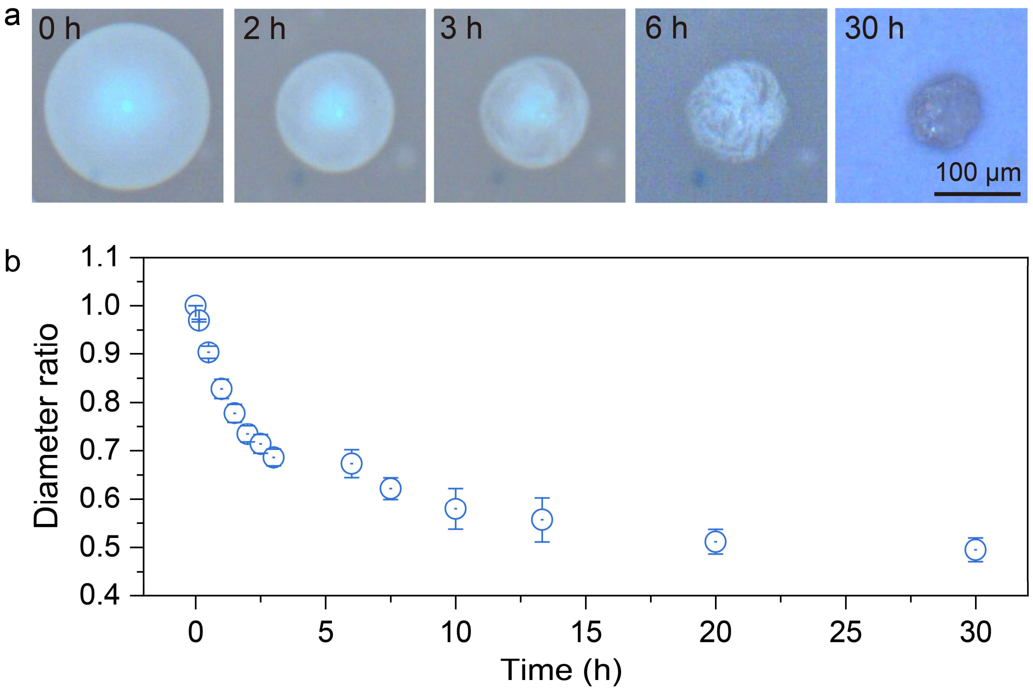

Figure S11. (a) Time-lapse series of microscopy images in reflection showing the evolution of a CNC microdroplet on the superamphiphobic surface during the drying process. The images were taken in brightfield. The loss of color in the dried microparticles arises from strong scattering at the buckled microparticle–air interface. **(b)** Diameter ratio of CNC microdroplets ([NaCl]/[CNC] = 100 μmol g^{-1}, TS = 0 s mL^{-1}) on the superamphiphobic surface over time. The drying process was conducted in an enclosed Petri dish with pure water used to regulate the relative humidity (RH = 100%).

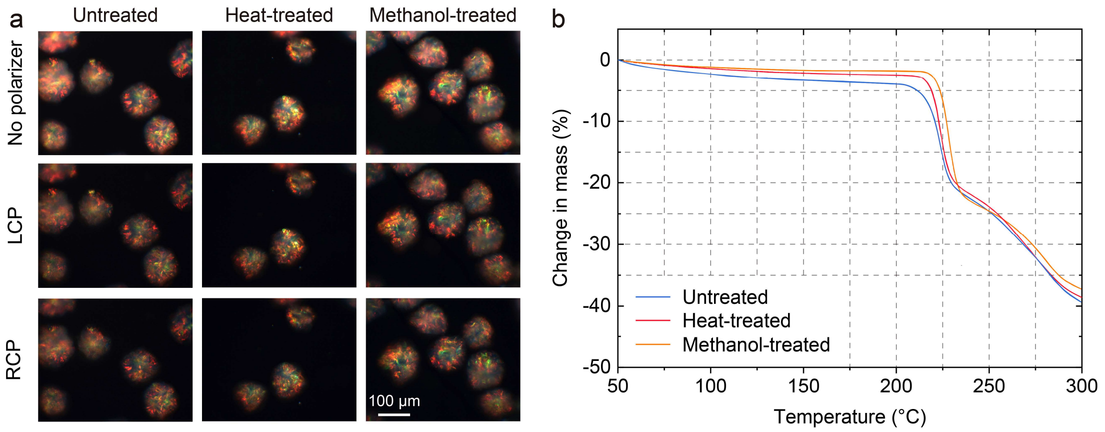

Figure S12. (a) Dark-field microscopy images in reflection of red CNC microparticles ([NaCl]/[CNC] = 50 µmol g^{-1}, TS = 6.67 s ml^{-1}) before and after heat-treatment (160 °C for 30 min) or methanol-treatment (immersion for 5 min). The microparticles were dispersed in an index-matching oil (n = 1.57) and imaged either without a filter or through an LCP or RCP filter. **(b)** Corresponding thermogravimetric analysis (TGA) of the untreated, heat-treated and methanol-treated red CNC microparticles.

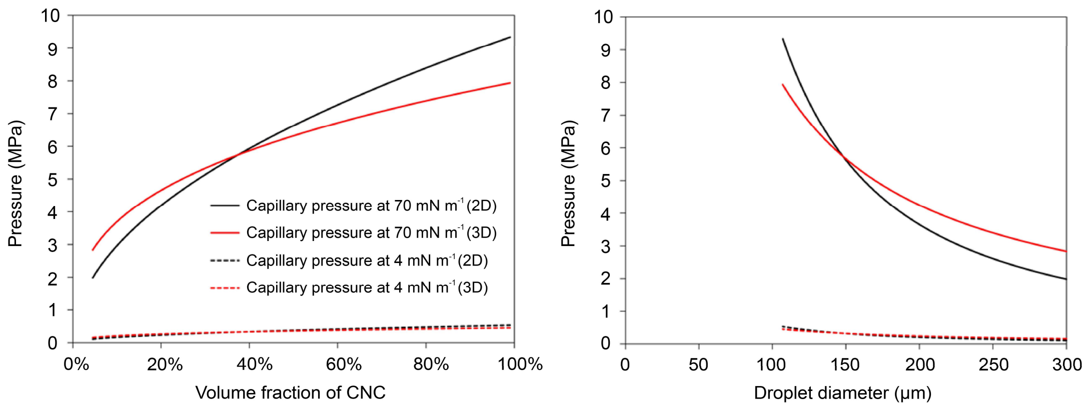

Figure S13. Capillary Laplace pressure calculated from the curvature of the Wigner-Seitz cells of individual CNCs using the cylindrical 2D model (black curves) or the spherical 3D model (red curves) for an initial concentration [CNC] = 7 wt% and diameter 300 µm and a surface tension corresponding to a water-air interface (70 mN/m for pure water, full lines) or for oil-water interface in presence of Span80 surfactant (estimated to ~4 mN/m, dashed lines). See Supplementary Discussion on page 2 of this document for further details.

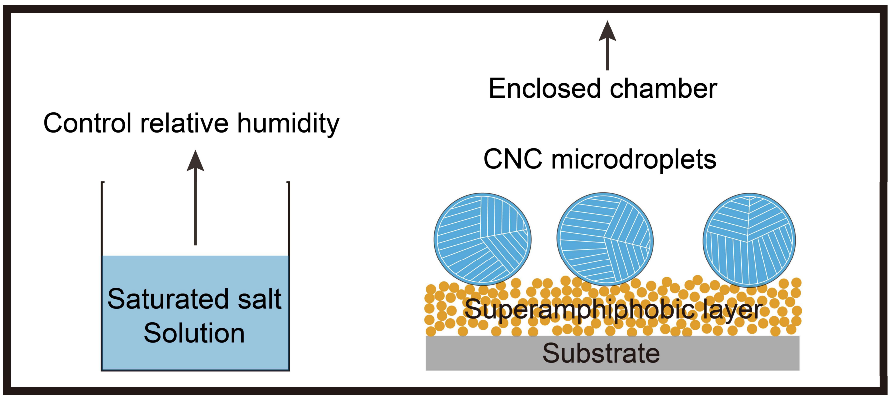

Figure S14. Schematic diagram of the setup for drying CNC microdroplets, where a saturated salt solution was used to lower the environmental relative humidity (RH).

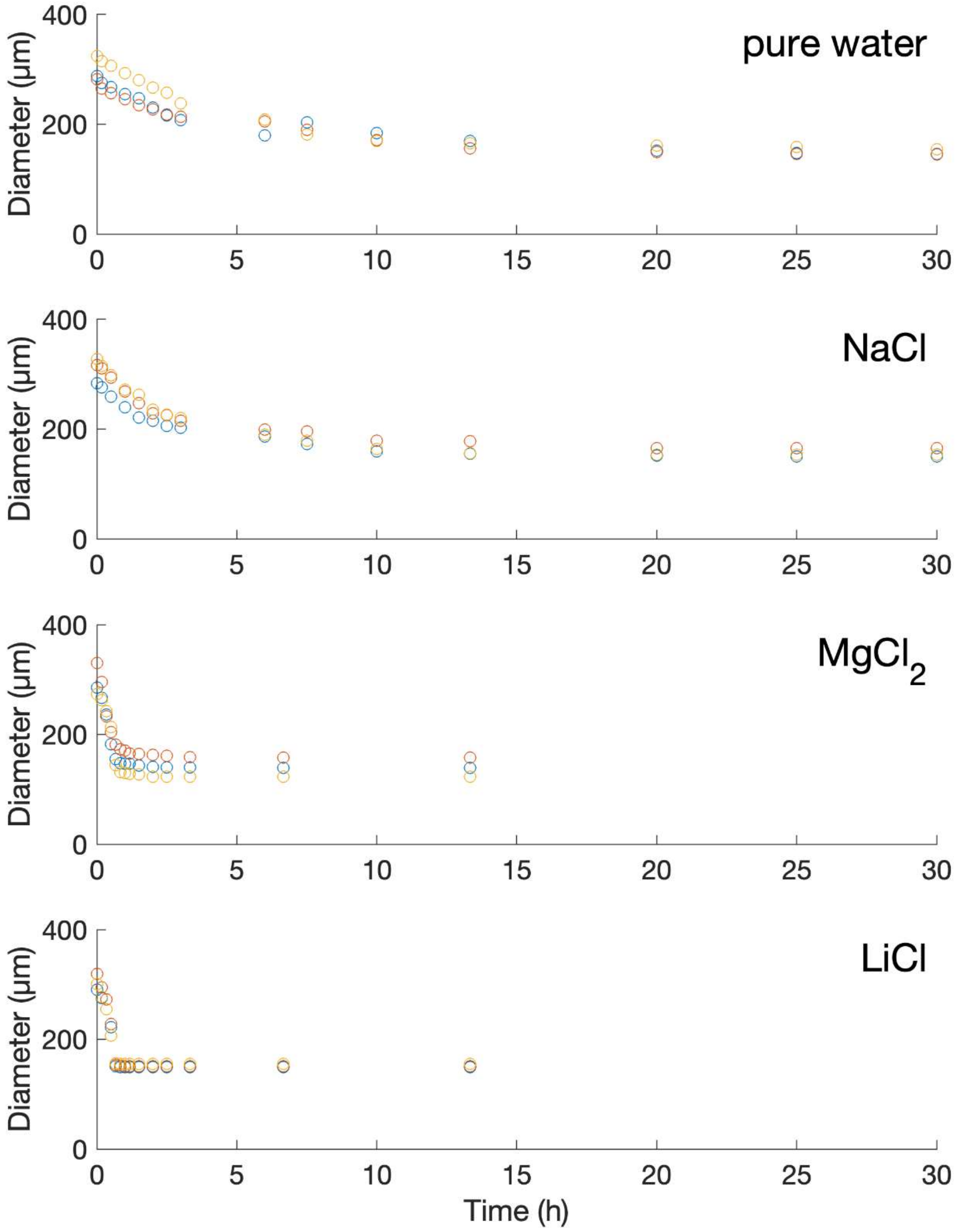

Figure S15. Evolution of the diameter (d) of CNC microdroplets over time under different relative humidity environments, as controlled with a pure water bath (RH = 100%) or a supersaturated solution of NaCl (RH = 76%), $MgCl_2$ (RH = 34%) or LiCl (RH = 12%). Datasets for three CNC microdroplets were plotted in different colors for each relative humidity condition.

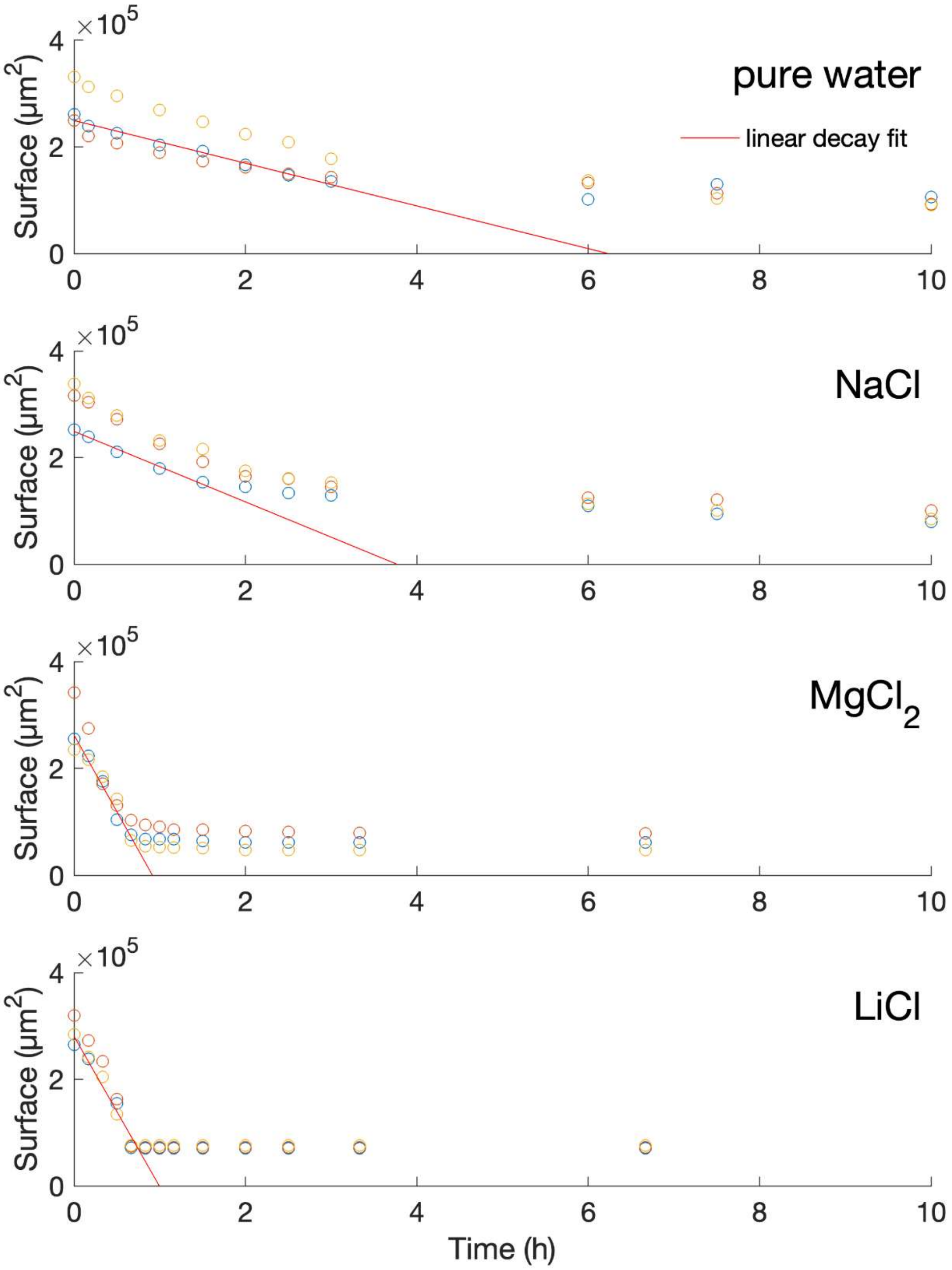

Figure S16. Evolution of the surface area ($s = 4\pi d^2$) of CNC microdroplets over time under different relative humidity environments, controlled with a pure water bath (RH = 100%) or a supersaturated solution of NaCl (RH = 76%), $MgCl_2$ (RH = 34%) or LiCl (RH = 12%). The final drying time t_d, which corresponds to the time when the surface area stopped evolving completely. The surface area (s) is considered to have stopped evolving completely when $\frac{[s(t)-s(t_{end})]}{[s(t=0)-s(t_{end})]} < 1\%$. $s(t = 0)$ and $s(t_{end})$ correspond to the surface area at the beginning and at the end of the drying process, respectively. In each case, a fitted curve (affine) for the initial drying profile of one of the droplets is highlighted in red. The intercept of the curve with the *x*-axis defines the idealized evaporation time t_{ev}, which corresponds to the evaporation time of a water droplet of the same initial size.

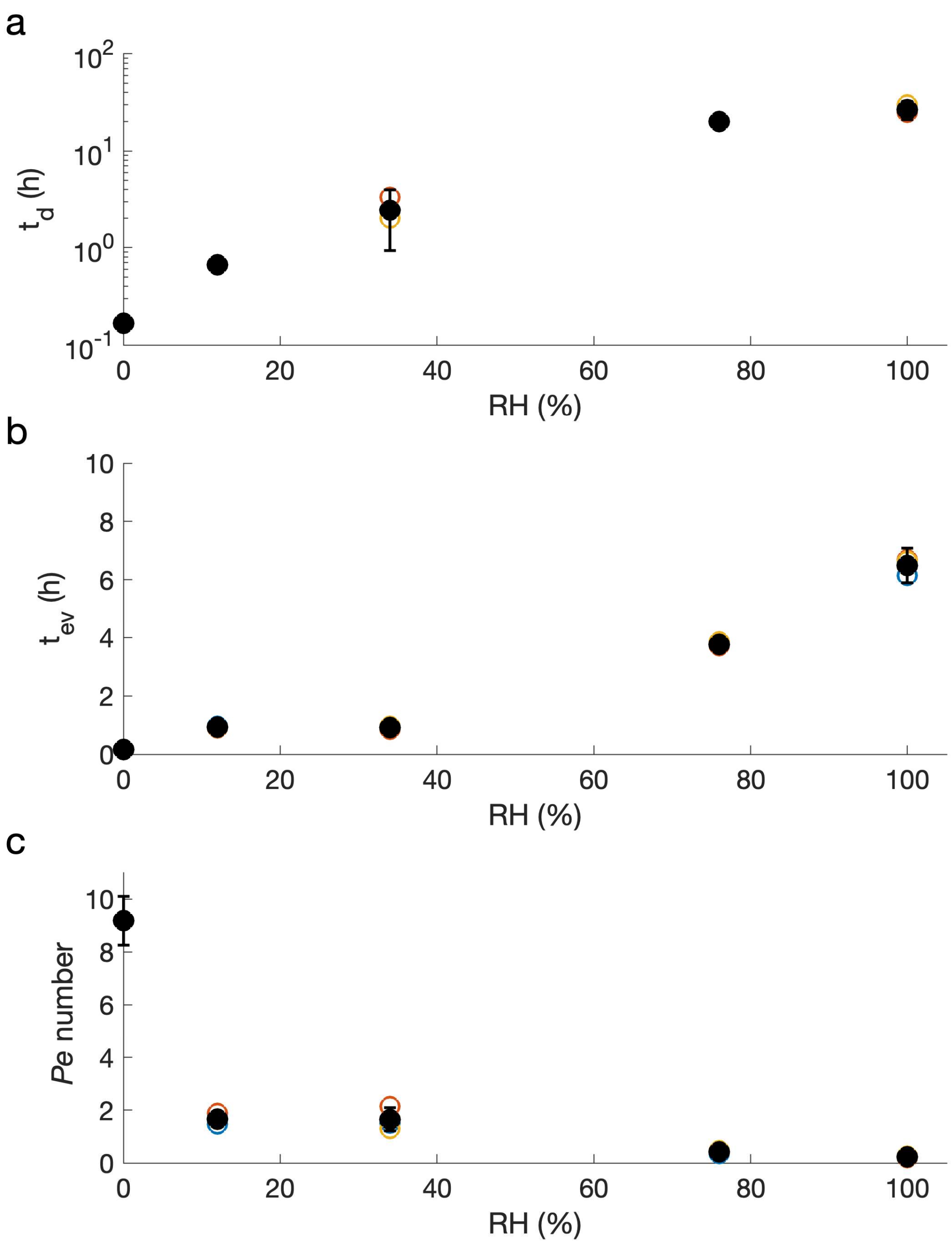

Figure S17. (a) The final drying time t_d, **(b)** idealized evaporation time t_{ev} and **(c)** corresponding Peclet number Pe for each drying condition, set by the choice of the supersaturated solution. The points at RH = 100% corresponds to pure water, while the point at RH ≈ 0% corresponds to drying under vacuum.

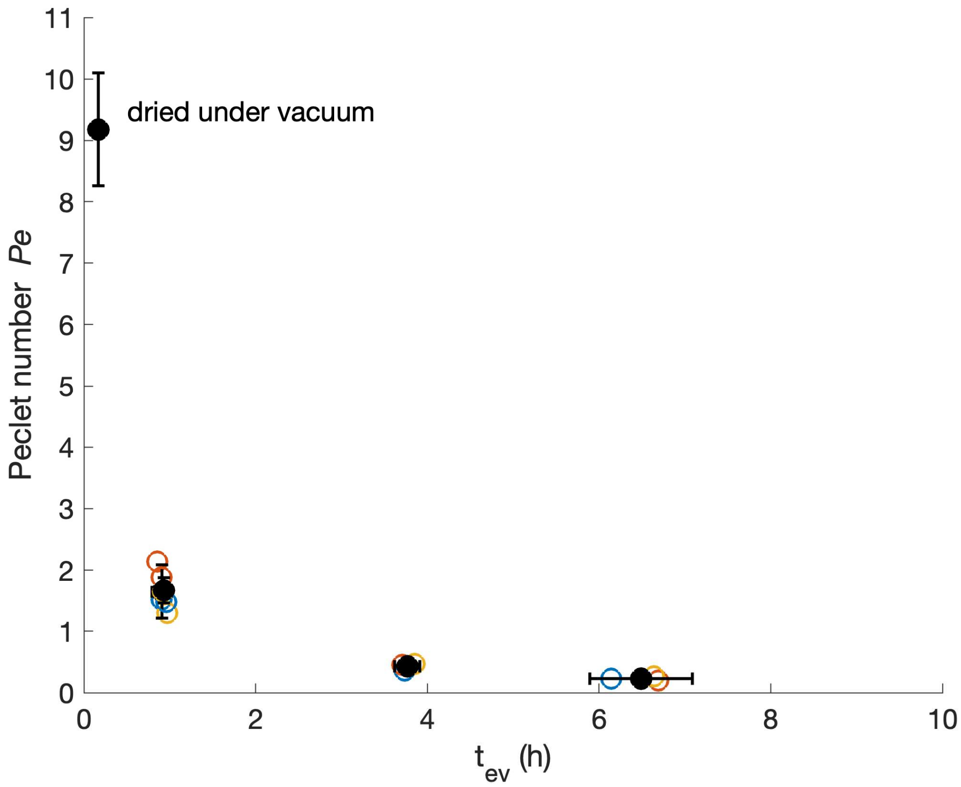

Figure S18. A plot of Peclet number Pe as a function of the evaporation time t_{ev}, which highlights the much higher Peclet number for the microparticles dried under vacuum (that display diminished optical properties). The Peclet number was defined as $Pe = t_D/t_{ev}$, where the diffusion time of the CNCs is estimated as $t_D = r_0^2/D_{\mathrm{CNC}}$, with r_0 the initial radius of the droplet and $D_{\mathrm{CNC}} = 4.14 \times 10^{-12}$ $\mathrm{m^2\,s^{-1}}$ is the effective diffusion coefficient of CNCs in water measured by DLS.

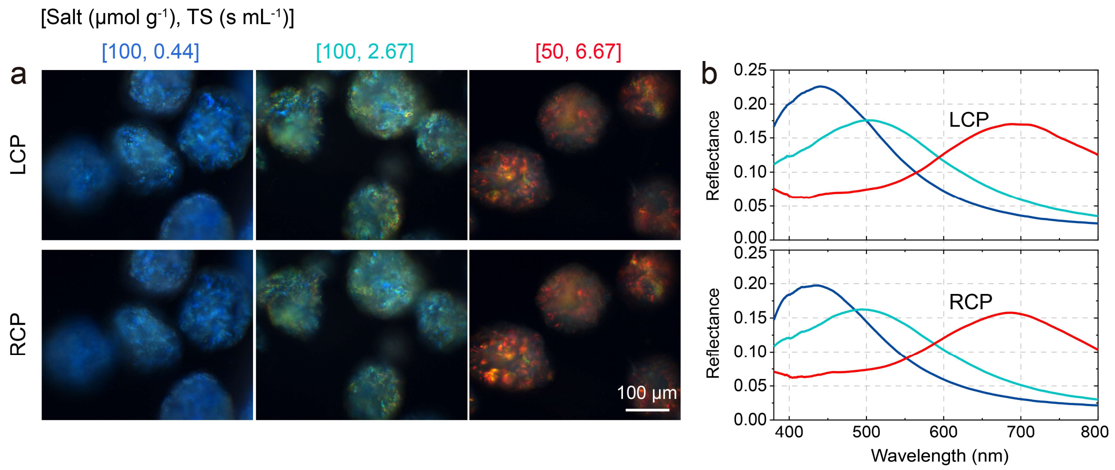

Figure S19. (a) Dark-field microscopy images in reflection of representative blue, cyan-green and red CNC microparticles dried at RH = 12% (drying time: ~40 min). **(b)** Corresponding micro-spectra of CNC microparticles in (a), averaged over 5+ locations. The microscope images and the corresponding micro-spectra were collected through either an LCP or RCP filter.

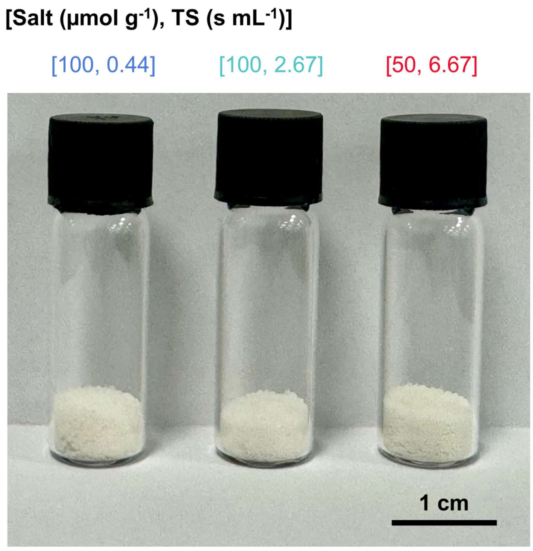

Figure S20. Dried CNC microparticles in glass bottles. The mass of blue ([NaCl]/[CNC] = 100 μmol g^{-1}, TS = 0.44 s mL^{-1}), cyan-green ([NaCl]/[CNC] = 50 μmol g^{-1}, TS = 2.67 s mL^{-1}), and red ([NaCl]/[CNC] = 50 μmol g^{-1}, TS = 6.67 s mL^{-1}) CNC microparticles dried at RH = 12% are 0.34 g, 0.30 g, and 0.36 g, respectively. White color of the dried microparticles arises from strong scattering at the buckled microparticle–air interface. Correct colors emerge when they are in an index-matching oil or PDMS. These pigments were used for the PDMS films on grass in Figure S27.

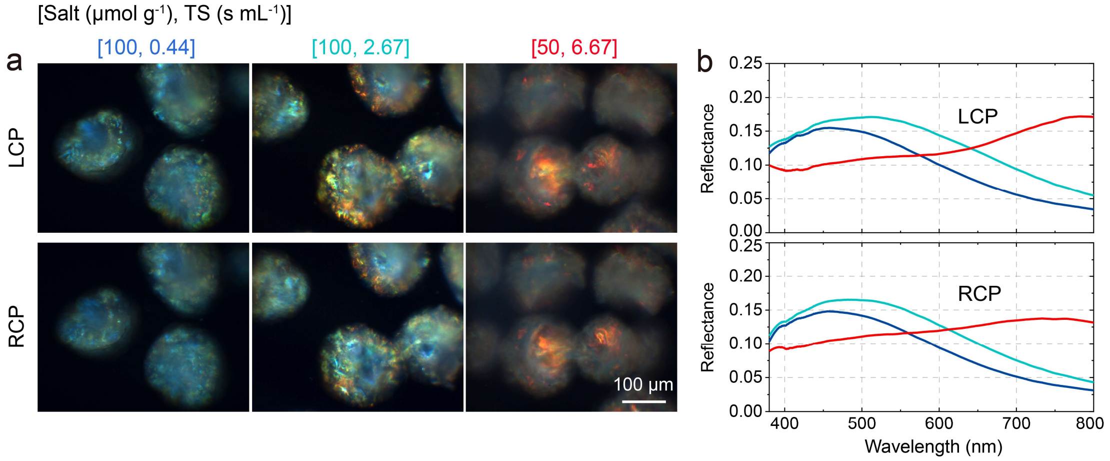

Figure S21. (a) Dark-field microscopy images in reflection of representative blue, cyan-green and red CNC microparticles dried under vacuum (drying time: ~10 min). **(b)** Corresponding micro-spectra of CNC microparticles in (a), averaged over 5+ locations. The microscope images and the corresponding micro-spectra were collected through either an LCP or RCP filter.

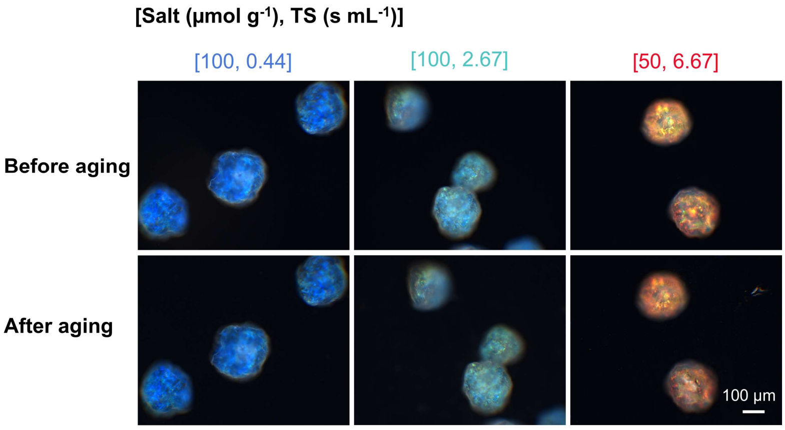

Figure S22. Dark-field microscopy images in reflection of blue, cyan-green and red CNC microparticles dried at RH = 12% in PDMS before (top) and after (bottom) UV aging.

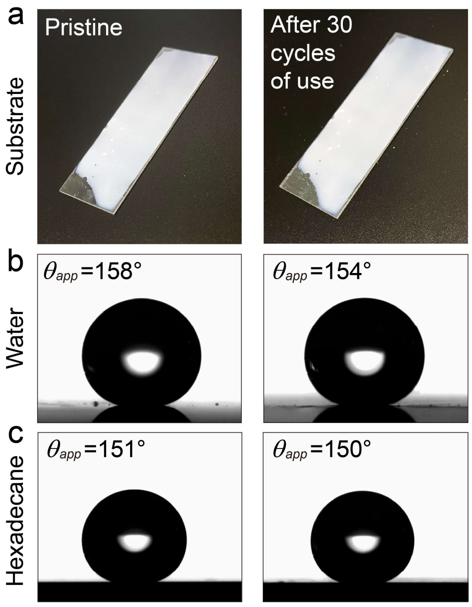

Figure S23. (a) The appearance of a pristine superamphiphobic substrate and the same substrate after 30 cycles of spraying CNC microdroplets and peeling off CNC microparticles. **(b,c)** 5 μL drops of (b) water and (c) hexadecane on the pristine superamphiphobic surface and the one after 30 cycles of CNC microparticle production, showing similar contact angles.

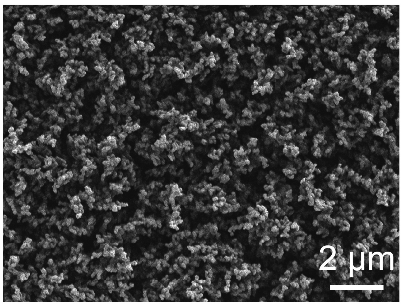

Figure S24. SEM image of the superamphiphobic surface, **(a)** before and **(b)** after 30 cycles of CNC microparticle production, showing the similarly intact micro-nano fractal structure.

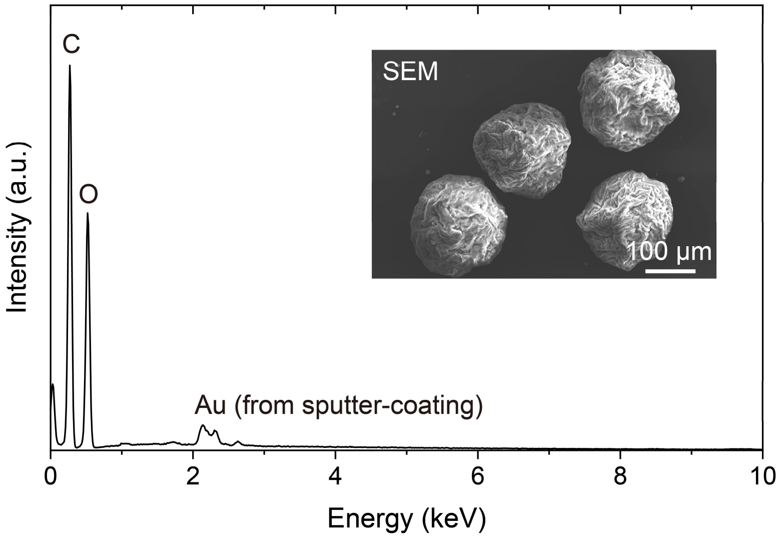

Figure S25. EDS analysis of CNC microparticles prepared on the superamphiphobic surface after 30 cycles of CNC microparticle production. Since the superamphiphobic coating is fluorinated, the elemental composition of F could be used as an indicator of whether the CNC microparticles are contaminated by the substrate. The EDS result shows no peak of F element on CNC microparticles. Au peaks are attributed to sputter-coating during sample preparation.

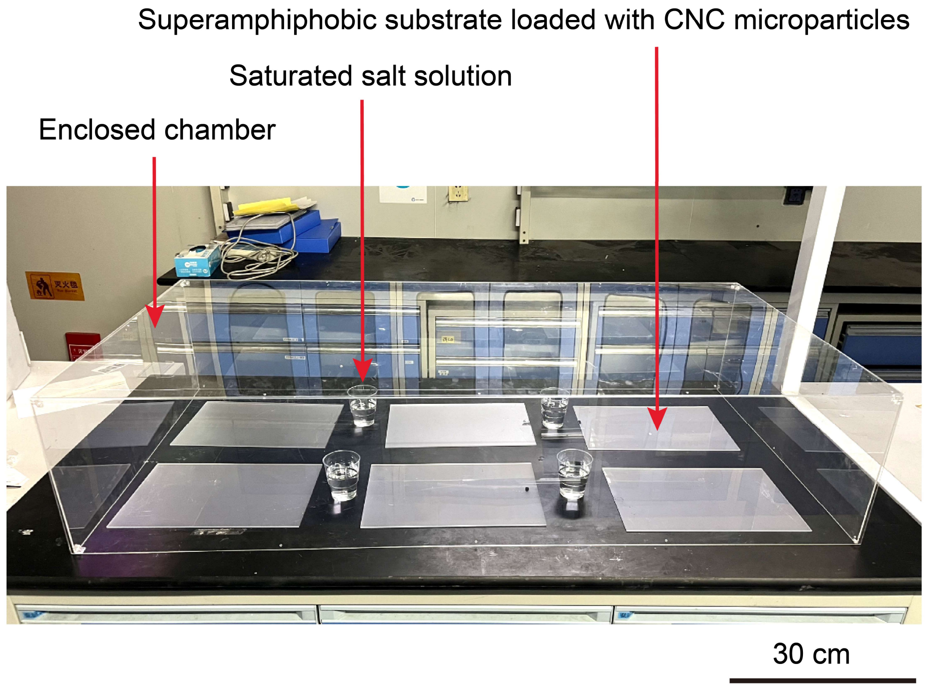

Figure S26. Demonstration of upscaled fabrication of the CNC photonic pigments, where each of the six superamphiphobic substrate has an area of 20 × 30 cm^2. Each substrate could produce approximately 0.19 g CNC photonic pigments per cycle. Therefore, six substrates could produce about 1.14 g per cycle.

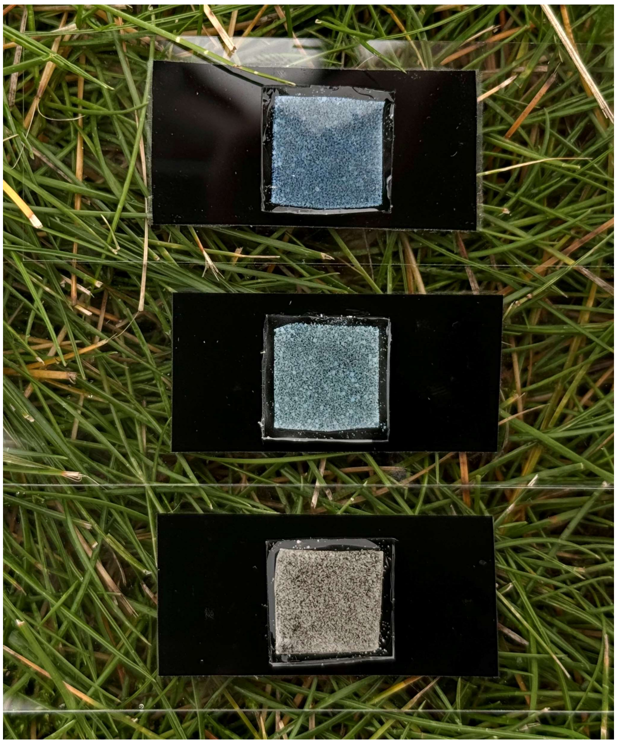

Figure S27. A macroscopic photo of CNC photonic pigments in a PDMS matrix. 40 mg of prepared blue, cyan-green, and red CNC pigments were respectively mixed with 150 mg of uncured polydimethylsiloxane (PDMS) and a small amount of carbon black (<0.1 mg) to cover an area of 1.44 cm^2 on glass slides. Carbon black was added into the binder matrix to absorb unwanted scattering at the buckled microparticle–PDMS interface. Blue, cyan-green, and red CNC pigments were prepared by drying at RH = 12%. Black tape was stuck to the bottom surface of these glass slides to get a black background. The photo was taken by an Apple iPhone 15 Pro smartphone.

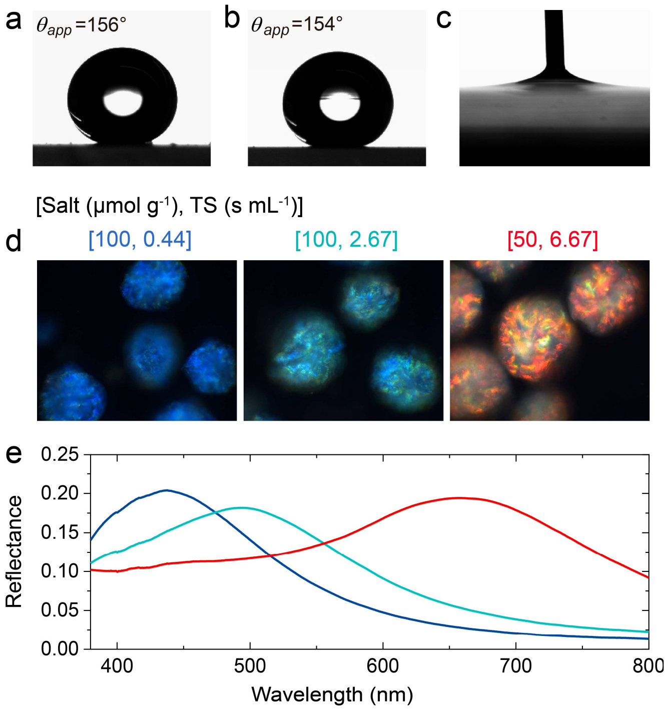

Figure S28. Dark-field microscope images in reflection of CNC microparticles dispersed in an index-matching oil (Cargille Series A, $n = 1.57$) prepared on superhydrophobic surfaces made by using commercial Ultra-Ever Dry paint. Side-view of a 5 μL drop of **(a)** water and **(b)** CNC suspension ([CNC] = 7.0 wt.%) on the superhydrophobic surfaces highlighting the apparent contact angle θ_{app}. **(c)** Side-view of a 5 μL drop of hexadecane on the surface. Hexadecane wetted the surface. The middle dark line is the needle for adding droplets. **(d)** Dark-field microscopy images in reflection of representative blue, cyan-green and red CNC microparticles prepared on the superhydrophobic surfaces (dried at RH = 12%). **(e)** Corresponding micro-spectra of CNC microparticles in (d), averaged over 4+ locations. The microscope images and the corresponding micro-spectra were collected without a filter.

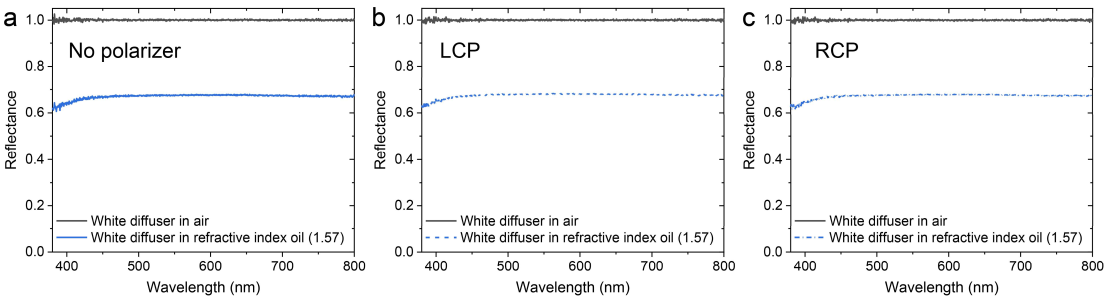

Figure S29. Reflectance of a white Lambertian diffuser under dark-field illumination either in air or coated with a thin layer of refractive index oil (n = 1.57). The spectra were collected either without a filter or through an LCP or RCP filter. The spectra are normalized to a white Lambertian diffuser in air, showing the reduced scattering when measured with a layer of oil. All spectra of CNC microparticles in this study were normalized to a white Lambertian diffuser measured coated with the refractive index oil.

References

1. R. K. O. Apenten, Q. H. Zhu, *Food Hydrocolloids*, 1996, **10**, 27-30.
2. D. Wang, Q. Sun, M. J. Hokkanen, C. Zhang, F.-Y. Lin, Q. Liu, S.-P. Zhu, T. Zhou, Q. Chang, B. He, Q. Zhou, L. Chen, Z. Wang, R. H. A. Ras and X. Deng, *Nature*, 2020, **582**, 55-59.
3. T. L. Liu and C.-J. C. J. Kim, *Science*, 2014, **346**, 1096-1100.
